# Exploring the Origin of Molecular Chirality: A Standalone Suite of Tools to Visualize and Analyze Transition Current Density

**DOI:** 10.1021/acs.jctc.6c00980

**Published:** 2026-06-24

**Authors:** Alberto Barlini, Marco Fusè, Julien Bloino

**Affiliations:** † 19004 Scuola Normale Superiore, Piazza dei Cavalieri 7, Pisa 56126, Italy; ‡ Dipartimento di Medicina Molecolare e Traslazionale, Università degli Studi di Brescia, Viale Europa 11, Brescia 25123, Italy

## Abstract

In this paper, we present a new software suite dedicated to the study and analysis of chiroptical signals and their nature. Beyond the simple evaluation of dipolar moments associated with either electronic or vibrational transitions, the tools it provides are able to compute and represent transition current densities (TCDs), which are directly connected to the electronic contribution to dipole transition moments. By mapping the corresponding vector fields into the real space, details on the sign and magnitude of the rotational strengths can be revealed in terms of localized current patterns on atoms, bonds, and functional groups, offering a unique view into the origins of chiroptical signatures. The software, open-source, is composed of two units: one to generate the fields from standard electronic structure calculations, and a visualization library that supports different representations to highlight, in the most suitable way, the influence of local regions in the systems under study on the overall chiral response. The capabilities of these tools are illustrated through a variety of cases, considering organic and organometallic molecules, closed- and open-shell species, under electronic and vibrational transitions. By making TCD analysis routine for a large range of molecular systems, this suite bridges the gap between black-box simulations of electronic and vibrational circular dichroism and chemically intuitive interpretations of molecular chirality.

## Graphical abstract

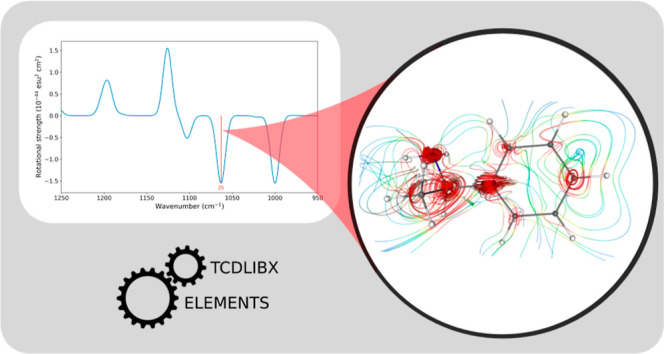

## 1 Introduction

Circular dichroism (CD), in its original, electronic (CD or now more commonly ECD), and vibrational (VCD) forms, is now a well-established technique to probe the structure and optical properties of chiral systems. While ECD can be used to distinguish enantiomers based on their absolute configuration, and therefore can play a crucial role in the determination of bioactive compounds for pharmaceutical purposes, for example,^1–3^ its dependence on the presence of chromophores and the typically limited number of observable bands impede considerably its applicability. For this reason, it is often complemented or supplanted by other chiral spectroscopies, in particular VCD. By probing chiroptical responses in the vibrational region, VCD can provide structural information, and thus has become an invaluable technique for investigating molecular chirality, as well as conformational ensembles and populations.^4–10^ Apart from systems where the spatial arrangement of chromophores allows the application of simple models such as exciton coupling to define molecular chirality^11^—an approach that can also be extended to specific vibrational transitions^12,13^ —the intrinsic complexity of the VCD and ECD signals recorded in most cases prevents experimental chiroptical spectra from being interpreted only on the basis of empirical rules.^5,14,15^ The advent of density functional theory (DFT) has greatly contributed to the field of chiroptical spectroscopy, enabling the routine analysis and interpretation of recorded spectra. With the support of computations, VCD has become a powerful tool for the assignment of absolute configuration and the investigation of conformational properties in a broad variety of molecular systems,^16–23^ including organometallic complexes.^19,21,24–32^ Foundational overviews and best-practice guides, spanning applications, conformational analysis, and implementations, are now well established and cover both theoretical and experimental aspects of the technique.^6,14,33–42^

The simple comparison of calculated and experimental global descriptors, such as the magnitude and sign of VCD difference intensities, can be sufficient to distinguish two systems, but it provides little information about the true origin of the chiroptical signal. As the structural complexity of chiral compounds increases and the role of the environment raises, as is the case in biological conditions for instance, a simple picture based on atomic polar and axial tensors and the visualization of vibrational modes becomes too simplistic. For this reason, in parallel to the development of theoretical models to predict chiroptical spectra, new visualization techniques and signal-partitioning approaches designed to guide the analysis and interpretation of experimental data have emerged. These tools help identify the different sources of chirality present in the molecular system under study and relate the observed response to the nature of the molecular groups composing it.^43–50^

A unifying route to mechanistic understanding is offered by the analysis of the transition current density (TCD), associated with electronic excitations, and vibrational transition current density (VTCD), associated with vibrations, which map the current-density patterns underlying the electric and magnetic transition dipole moments that drive chiroptical responses. Philosophically connected to the early attempts to compute VCD,^51^ the TCD formalism was introduced by Nafie^43^ and first applied on electronic excitations.^45^ The same current-density viewpoint extends naturally to the vibrational case, in which VTCD has been used to analyze VCD transitions.^44,52^ More recently, this approach has been consolidated and extended into a practical simulation and visualization framework.^53^ However, this original proof of concept suffered from several major limitations in terms of code availability, interoperability, and extensibility.

Here, a new implementation for the calculation of TCD and VTCD fields is proposed in a fully open-source library system written in Fortran, ELEMENTS. The library is modular and reusable and offers standardized interfaces for parsing data from general-purpose quantum chemical programs. Starting from electronic density calculations, it provides all necessary computing tools to construct TCD and VTCD grids and export volumetric data for visualization. This approach can help link spectral features to their underlying electronic phenomena by computing TCDs directly from standard quantum-chemical calculations. The result is a unified framework for exploring molecular chirality across a broad range of chemical systems.

For the 2D and 3D visualization of the current density data on top of the molecular structure, and the graphical analysis of the local contributions from atomic groups to the chiral response, ELEMENTS is complemented by a dedicated processing library, TCDLIBX. Whereas previous stand-alone tools such as VCDtools^54^ focused primarily on spectral analysis, TCDLIBX extends those capabilities to visualize TCDs and VTCDs and to process volumetric vector-field data sets generated by the ELEMENTS computational core.

In the following sections, we first summarize the theoretical background used to compute the quantities necessary for the prediction of ECD and VCD spectra, and how they relate to TCD and VTCD. We then detail the implementation strategies in ELEMENTS, show applications of the newly developed toolbox on representative molecular systems, and illustrate how this integrated approach enhances both theoretical understanding and practical interpretation of molecular chirality.

## 2 Theoretical Background

For an electronic excitation |φ_0_⟩ → |φ_*n*_⟩ of frequency ω_0*n*_, the ECD intensity is proportional to the rotational strength, which, in the length-gauge representation, is written as

1 $$R_{0n}^{ECD}=Im\left[\mu_{0n}^{el}\cdot m_{n0}^{el}\right]$$

where $\mu_{0n}^{el}=-\left\langle \varphi_{0}\left|r\right|\varphi_{n}\right\rangle$ and $m_{n0}^{el}=-\frac{1}{2}\left\langle \varphi_{n}\left|r\times p\right|\varphi_{0}\right\rangle$ , with **p** = −*i*∇, are the electric- and magnetic-dipole transition moments. In the velocity-gauge formulation, eq 2 is written as

2 $$R_{0n}^{ECD}=\frac{1}{\omega_{0n}}Re\left[\mu_{0n}^{el}\cdot m_{n0}^{el}\right]$$

where the relation −*i*ω_0*n*_⟨φ_0_|**r**|φ_*n*_⟩ = ⟨φ_0_|**p**|φ_*n*_⟩ has been employed to express the electric–dipole transition moment in the velocity representation. All quantities are expressed in atomic units. The velocity-gauge representation of the electric transition moment will be used for the remainder of this manuscript. The rotational strength in eq 2 is a global quantity and contains no information about the spatial origin of the chiroptical response. To access this local information, we switch to the TCD formalism.^43^ Following Nafie and co-workers,^43,55^ we define the TCD associated with the |φ_0_⟩ → |φ_*n*_⟩ electronic excitation

3 $$j_{0n}\left(r\right)=\left\langle \varphi_{0}\left|\nabla \left(r\right)\right|\varphi_{n}\right\rangle .$$

In practice, eq 3 is evaluated in the atomic orbital basis as^43^

4 $$j_{0n}\left(r\right)=\sum_{\mu \nu }\left(T_{\mu \nu }^{0n}-T_{\nu \mu }^{0n}\right)\chi_{\mu }\left(r\right)\nabla \chi_{\nu }\left(r\right)$$

where $T_{\mu \nu }^{0n}$ are the elements of the transition density matrix, which in the present work is obtained from time-dependent density functional theory (TD-DFT) calculations, and χ_μ_(**r**) is an atomic orbital basis function evaluated at point **r**. In addition, the angular TCD is defined as^43,53^

5 $$j_{0n}^{ang}\left(r\right)=\frac{1}{2}r\times j_{0n}\left(r\right)$$

These TCDs are related to the electric and magnetic dipole transition moments through^43^

6 $$\mu_{0n}^{el}=-i\int j_{0n}\left(r\right)dr$$

7 $$m_{n0}^{el}=\frac{i}{2}\int r\times j_{n0}\left(r\right)dr=i\int j_{n0}^{ang}\left(r\right)dr$$

and provide spatial information about the origin of the electronic transitions in ECD spectra.

A similar approach can be followed for VCD. Let us consider a vibrational transition |χ_0_⟩ → |χ_*k*_⟩ of frequency ω_0*k*_. The rotational strength in the velocity gauge is expressed analogously as^55^

8 $$R_{0k}^{VCD}=\frac{1}{\omega_{0k}}Re\left[\mu_{0k}^{tot}\cdot m_{k0}^{tot}\right]$$

where **μ**_0*k*_^tot^ = **μ**_0*k*_^el^ + **μ**_0*k*_^nuc^ and **m**_*k*0_^tot^ = **m**_*k*0_^el^ + **m**_*k*0_^nuc^ are the total electric and magnetic vibrational transition dipole moments, including both electronic and nuclear contributions. In the following, we will limit ourselves to fundamental transitions within the harmonic-oscillator approximation. Extension to the anharmonic description and the associated challenges have been detailed in ref 53.

Within this framework, several formulations of the pure electronic contributions to the vibrational transition moments of the electric and magnetic dipoles have been proposed.^55–58^ In this work, we adopt the vibronic coupling theory (VCT) described in refs 53 and 56 . In VCT, transition moments are expressed in terms of the vibrational transition current density (VTCD), **J**_0*k*_(**r**), as^43,44,52,55^

9 $$\mu_{0k}^{el}=-i\sqrt{\frac{\omega_{0k}}{2}}\int J_{0k}\left(r\right)dr$$

10 $$m_{k0}^{el}=-i\sqrt{2\omega_{0k}}\int r\times J_{k0}\left(r\right)dr=-i\sqrt{2\omega_{0k}}\int J_{k0}^{ang}\left(r\right)dr$$

with the VTCD given by^59^

11 J0k(r)=∑n>0∑M⟨φ0|∇(r)|φn⟩ω0n⟨φn|∂∂RM|φ0⟩SMk=∑n>0j0n(r)ω0nD0n,k

and where the angular VTCD is^44,52,59^

12 $$J_{k0}^{ang}\left(r\right)=r\times J_{k0}\left(r\right)$$

Here, **S**_*Mk*_ = ∂**R**_*M*_/∂*Q*_*k*_ is the nuclear displacement vector of nucleus *M* along the *k*-th mass-weighted normal coordinate *Q*_*k*_, and *D*_0*n*,*k*_ denotes the nonadiabatic coupling (NAC) between the ground and excited electronic states along mode *k*. The total vibrational transition moments are obtained by adding the pure nuclear contributions^55,59^

13 $$\mu_{0k}^{nuc}=i\sqrt{\frac{\omega_{0k}}{2}}\sum_{M}Z_{M}S_{Mk}$$

14 $$m_{0k}^{nuc}=i\sqrt{2\omega_{0k}}\sum_{M}Z_{M}\left(R_{M}\times S_{Mk}\right)$$

In analogy with TCD, the VTCD can be evaluated as

15 J0k(r)=∑n>0∑μν(Tνμ0n−Tμν0n)χμ(r)∇χν(r)ω0nD0n,k=∑n>0j0n(r)ω0nD0n,k

Note that eq 15 mirrors eq 11, which is expressed in terms of exact wave functions. Therefore, the VTCD can be interpreted as a vibronically weighted superposition of electronic TCDs, where each electronic transition contributes according to its NAC with the selected vibrational mode and is damped by the corresponding excitation energy. The corresponding VTCD maps capture the current induced by nuclear motion and link band signs and intensities to spatially localized current patterns.

It is important to note that the molecular properties related to ECD and VCD are, in principle, independent of the gauge used to describe the interaction with the electromagnetic field.^60–62^ For exact wave functions, the rotational strengths are invariant with respect to both the choice of gauge origin and the representation (length or velocity form) of the electric dipole operator.^58,63^ However, in practical calculations with finite one-electron basis sets, this gauge invariance is only approximate, and different representations may lead to numerical differences. In the formulation chosen here, TCD and VTCD are obtained in the velocity representation of the electric dipole operator. Alternatively, our implementation also allows the computation of the length-gauge transition dipole density,^43^ thereby providing the length-gauge electric dipole directly. In the following, only the velocity-gauge will be considered since the corresponding TCD and VTCD maps provide conveniently a gauge-independent real-space decomposition of the related rotational strengths.

As a final remark, the term EDTM will be used to refer to the transition moments of the electric dipole in general, be it electronic (ECD) or vibrational (VCD), and MDTM for that of the magnetic dipole.

## 3 Implementation Details

Before considering study cases on the use of transition current densities, let us detail some primary aspects of the implementation, the standard usage workflow, and the core capabilities of the ELEMENTS library in connection with the construction of the TCD and VTCD vector fields, together with the dedicated TCDLIBX visualization package for postprocessing and rendering TCD analyses. The libraries include user programs, one for the generation of the TCD fields (GENTCD) and one for VTCD (GENVTCD), packaged with ELEMENTS, and TCDVIS, a graphical user interface for TCDLIBX. The latter uses the ESTAMPES Python library,^64^ to load molecular data from quantum mechanical calculations.

### 3.1 Workflow for ELEMENTS

For TCD, the implementation follows a modular workflow (Figure 1) in which molecular data are first read from external chemical calculation output files and stored internally. Pure basis sets are converted to their Cartesian representation through dedicated routines implemented in the library. The current density is then constructed on a grid before finally being written into so-called cube files. In its current implementation, the program requires a GAUSSIAN formatted checkpoint file^65^ containing molecular data together with excited-state transition densities computed using TD-DFT. It should be noted that, thanks to its interface system, the library can be straightforwardly extended to support any quantum chemical package providing the necessary quantities. The code can handle both closed- and open-shell systems. The implementation provides two types of cube: the TCD defined in eq 4 and the angular TCD associated with the magnetic transition dipole moment of eq 5. TCD values are evaluated on regular cubic grids, whose parameters (origin, steps, number of points) can be read from an external file or can be generated automatically. When multiple electronic excitations are specified, the program can construct a single grid that encloses all states via a global bounding-box estimate, fit a grid to a designated state, or generate a dedicated grid on-the-fly for each state. The grid boundaries are estimated from the transition-density matrix associated with the selected electronic excitation. Starting from an atom-centered box defined by the nuclear coordinates and covalent radii, the code evaluates the TCD at the box vertices. The box is iteratively enlarged until the TCD norm falls below a fixed threshold of 10^–10^ atomic units at the boundary. For a common grid over several excitations, the final bounding box is obtained as the union of the state-specific boxes. For every grid, the origin, number of points, and spacing are reported. As consistency checks, the program can also compute the electronic component of the electric or magnetic dipole transition moments obtained by numerical integrations of the cubes.

**Figure 1:**
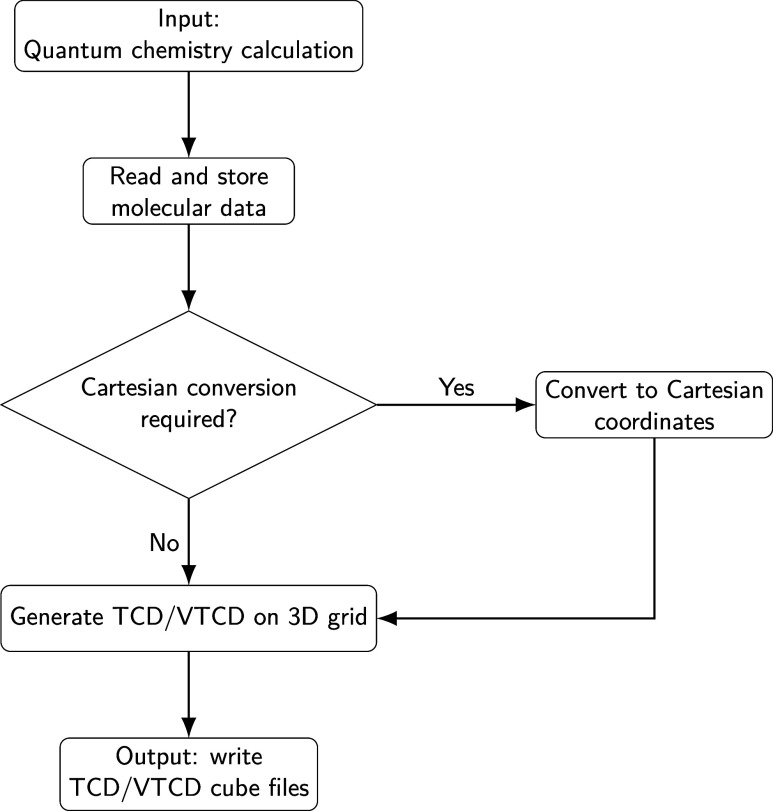
Schematic representation of the TCD/VTCD computational procedure.

**Figure 2:**
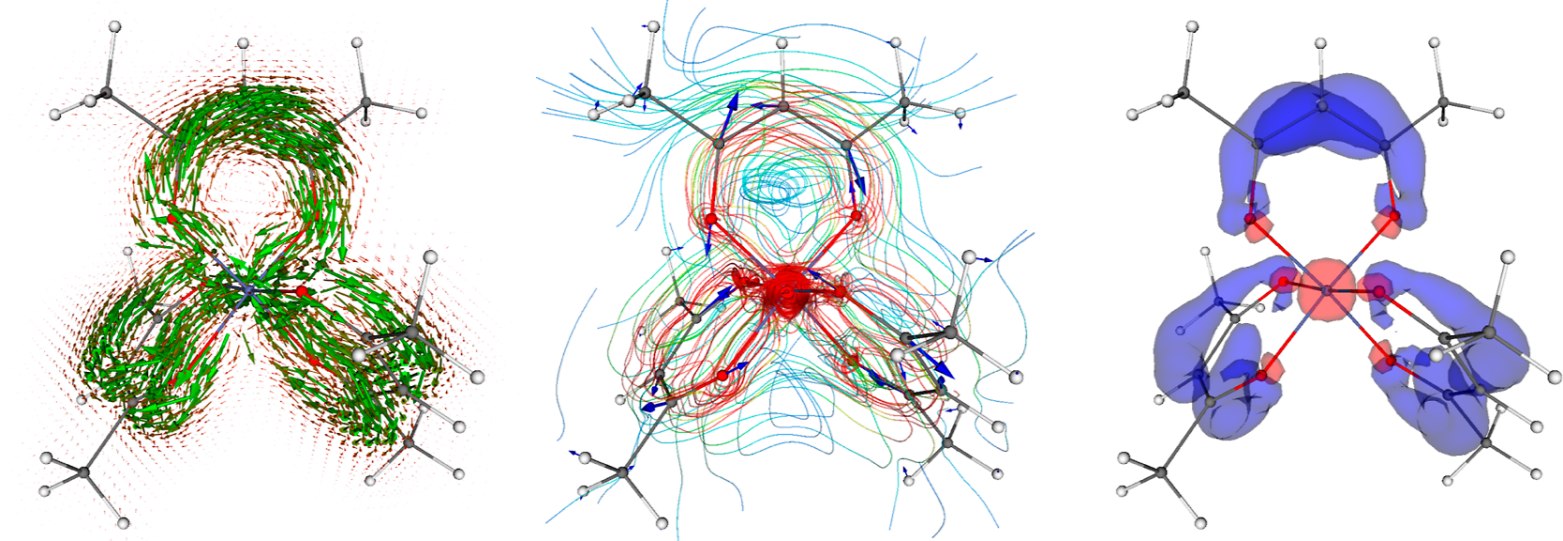
Example of graphical representations of **J**_0*k*_ with hedgehog (left) and streamlines representation (center). On the right, the *R* ^*m*^ scalar field connected to the same molecular vibration *k* is depicted. The normal mode is represented as blue arrows in the central panel.

For VTCD, the dedicated program, GENVTCD, requires additional information, namely the NACs and transition density matrix elements for all electronic states involved in the summation in eq 11, as well as vibrational frequencies and normal modes of the ground electronic state. The program checks that the molecule is in Eckart orientation and, if necessary, rotates the structure to ensure that the couplings between vibrations, rotations, and translations are minimal, applying the transformation to all relevant quantities. In analogy with TCD, two vector maps can be constructed: the VTCD defined in eq 15 and the corresponding angular VTCD associated with the electronic contribution to the vibrational magnetic transition dipole moment of eq 12.

The main execution flow mirrors that of GENTCD. The program begins by reading the checkpoint files to recover molecular data, excited-state information, and vibrational information. For the electronic transition-related information, since NACs are generally computed for a single electronic state at a time, the program supports a template system and can build the list of electronic excited states to include based on the files matching the template. The generation of the TCD elements uses the same routines as GENTCD. Since the computation of the contribution from each electronic transition moment can be expensive, it is possible to store each TCD cube data and the associated molecular data in a binary file to avoid restarting from the beginning when selecting a different normal mode to analyze.

Two computational pathways are thus available. When binary files are provided by the user, the program reloads the data and grid parameters from this file and, for each electronic state, combines the precomputed TCD cubes with the NAC vectors to construct the VTCD. In the fully generative mode, used when no precomputed cubes are available, the code first checks and, if necessary, performs the conversion from pure to Cartesian functions. The grid-generation strategy is the same as in the TCD module: the spatial grid is determined by reading a user-specified grid file, by constructing a grid tailored to a single reference state and then applying it to all others, or by building a unified grid that spans the spatial envelope of all selected states. Finally, the nuclear contributions to the electric and magnetic dipole moments can be computed upon request alongside their electronic counterparts for consistency checks.

### 3.2 Workflow for TCDLIBX

In the absence of a dedicated viewer for the visualization of vector fields in the context of molecular spectroscopy, we developed a library and a viewer based on VTK,^66^ taking advantage of its Python bindings, to provide simple and effective tools for the analysis and processing of data obtained with ELEMENTS. At present, the interface operates through text files containing information derived from quantum-mechanical calculations, such as GAUSSIAN formatted checkpoint (fchk) files, as well as files containing volumetric data in cube format. The extraction of the required information from the fchk files is carried out using the ESTAMPES library.^64^ The library can carry out different types of arithmetic operations between different data sets. While the performance is sufficient for standard applications like the ones considered in this work, an alternative path under development will be to directly offload such calculations to optimized routines built in ELEMENTS for the most demanding cases.

In addition to standard visualization capabilities—such as the representations of normal modes, transition dipoles, and scalar-field isosurfaces—the viewer provides two full vector-field visualization schemes. Following the work in ref 53, both hedgehog and streamlines visualizations are supported (see Figure 2).

Moreover, the viewer supports scalar-field representations of the rotational strength, defined as

16 $$R_{0n}^{\mu }\left(r\right)=\frac{1}{\omega_{0n}}Re\left[\mu_{0n}\left(r\right)\cdot m_{n0}\right]$$

17 $$R_{0n}^{m}\left(r\right)=\frac{1}{\omega_{0n}}Re\left[\mu_{0n}\cdot m_{n0}\left(r\right)\right]$$

where the scalar products are between the corresponding local and integrated dipole transition moments. Depending on the specific combination used, these maps provide insight into the dipole strength or the rotational strength of the transition under analysis.^5,53^

For streamlines generation, two seed-placement strategies have been implemented: uniform seeding within the rotational ellipsoid,^67^ and placement of seeds in proximity to atoms, excluding an inner region with a radius smaller than 10% of the van der Waals radius (see Figure S1 in the Supporting Information). To recover directional information—lost when transitioning from hedgehog to streamlines representations—animated seed propagation along streamlines can be enabled by the user.

Fragment information and space partitioning can also be activated to study the contribution of different moieties of the system to the overall transition intensity.

The library also provides scripts that can be used directly from the command line to generate two-dimensional representations of the vector field in hedgehog or streamlines formats. The represented vector field can be obtained either by integrating the field along the axis perpendicular to the displayed plane or by selecting a specific subportion of a user-defined slab. In panel (a) of Figure 3, the TCDVIS graphical interface is shown, illustrating the definition of a subspace. In the right panels of Figure 3 two maps are shown: one obtained by projecting along the entire *y* axis (b) and the other obtained by considering only the contributions lying in the front portion of the molecule (c). In the former case, the cancellation between front and rear contributions prevents the observation of the charge flow along the poly alkyne chains, which can be easily spotted in the latter instead.

**Figure 3:**
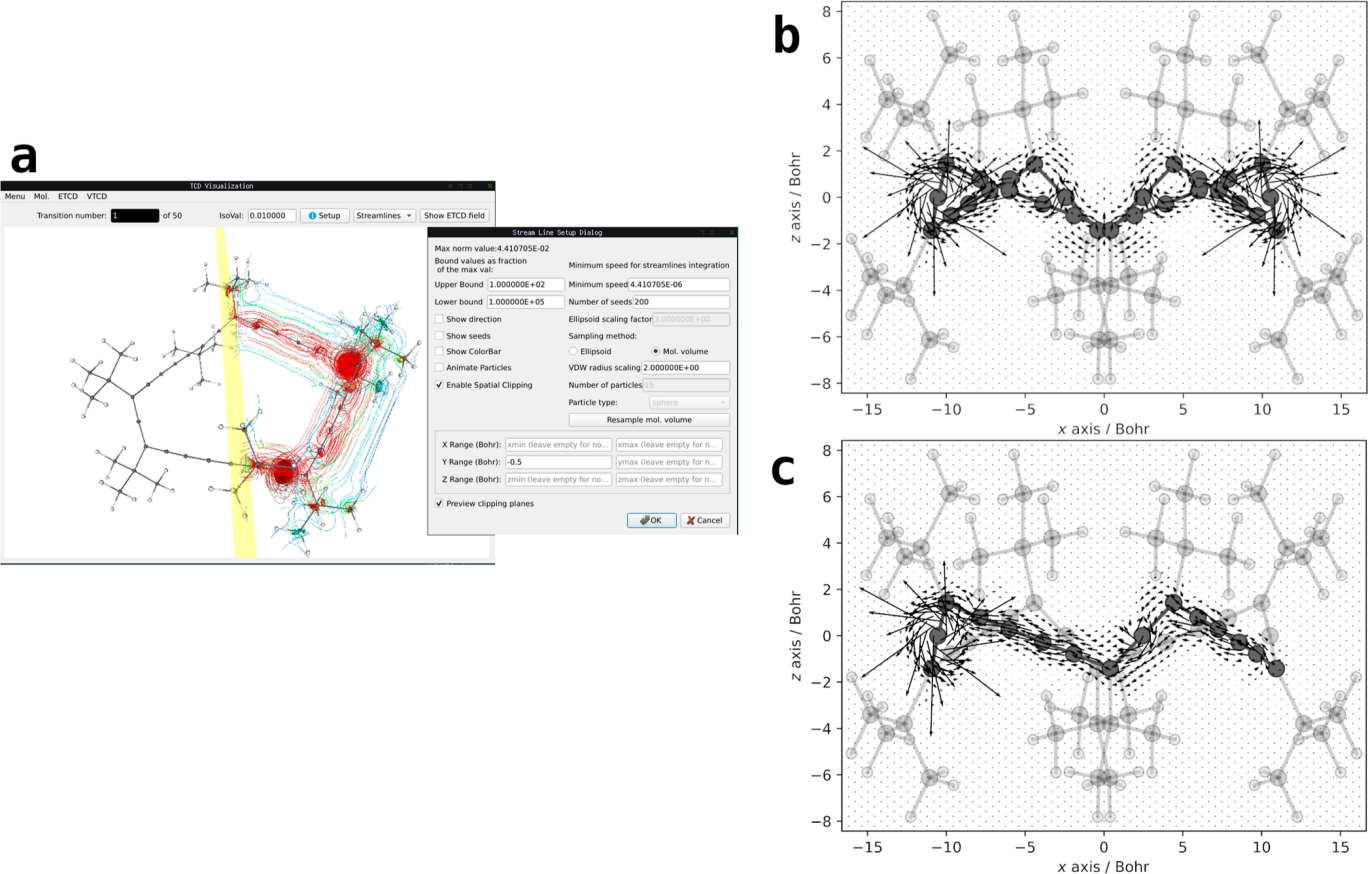
Panel (a) illustrates the three-dimensional representation of a subspace definition in TCDVIS. Panels (b,c) report the maps obtained by integrating along the full *y* axis and along a selected portion of it (*y* < – 0.5 Bohr), respectively. Atoms lying outside the integrated subspace are rendered as transparent.

## 4 Computational Details

All calculations presented in this work were carried out at the density functional theory (DFT) and time-dependent DFT (TD-DFT) levels of theory performed with the GAUSSIAN16 suite of quantum chemical programs.^65^ Whenever possible, the level of theory of the calculations was selected to mirror those adopted in the corresponding studies, thereby facilitating a more direct reproduction and interpretation of the reported data.

For the study of 2*S*-2-methylaziridine (Figure 4a), the CAM-B3LYP exchange correlation functional,^68^ in combination with the aug-cc-pVTZ basis set, was employed. The TCD cubes were generated according to eq 4 on a single grid, with the grid boundaries determined from the first five electronically excited states.

**Figure 4:**
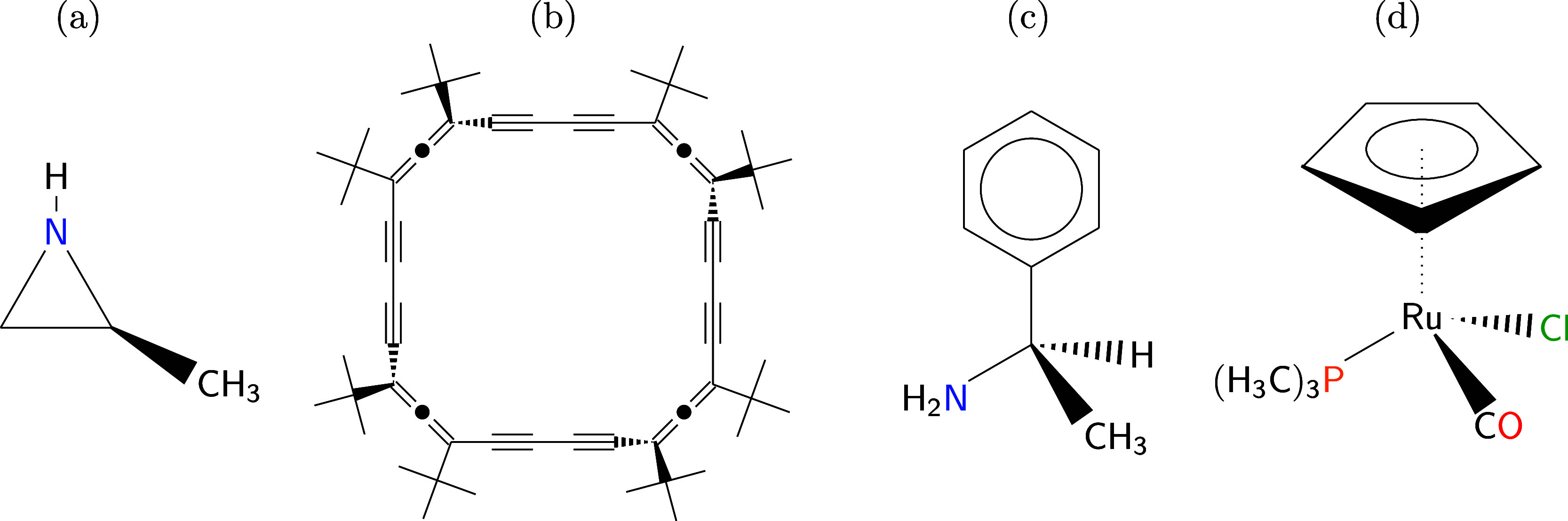
2D skeletal structures of the molecules considered in this study: (a) 2*S*-2-methylaziridine, (b) (P,P,P,P)-(−)-1 alleno acetylenic crown macrocycle, (c) (*R*)-α-phenylethylamine, (d) chloro­(carbonyl)­(trimethylphosphine)­(η^5^-cyclopentadienyl)­ruthenium­(II).

The alleno acetylenic crown macrocycle (P,P,P,P)-(−)-1 (Figure 4b) was treated with the same CAM-B3LYP functional^68^ and 6-31G­(d) basis set as in ref 69. In this case, we computed and analyzed the TCD and the magnetic moment cubes associated with the two lowest electronic transitions. The evaluation of each fragment’s contribution to the TCD was carried out according to the atoms-in-molecules (AIM) partitioning scheme using the MULTIWFN program.^70^

For (*R*)-α-phenylethylamine (PEA) (Figure 4c), we adopted the same level of theory as in ref 71, to which we refer for additional computational details. The VTCD cubes were generated according to eq 15 on a single grid with boundaries determined from the first 300 excited electronic states.

For the Ru­(II) complex (Figure 4d) previously investigated in ref 72, equilibrium geometries, harmonic vibrational frequencies, normal modes, and nonadiabatic couplings were obtained using the B3LYP functional.^73–75^ For the ruthenium atom, the LANL2DZ effective core potential and associated basis set were employed.^76^ For all the other atoms, the SNSD basis set was employed.^77,78^ Like for PEA, the VTCD cubes were generated according to eq 15 on a single grid. The boundaries were determined from the first 150 excited electronic states.

## 5 Results and Discussions

The results presented below are organized by spectroscopy, starting from ECD, for which the calculation of the EDTM and MDTM is straightforward, followed by VCD, which involves a summation over the manifold of excited electronic states.

### 5.1 Transition Current Densities for Electronic Circular Dichroism

Rauk showed that, for aziridines, the low-energy chiroptical response is governed predominantly by the nitrogen center, while the stereocenter on carbon plays a comparatively minor role, except possibly for the second transition.^79^ To confirm this hypothesis, we consider the first two singlet excitations of the two diastereomers (1*S*,2*S*)-2-methylaziridine (shortened to 1S) and (1*R*,2*S*)-2-methylaziridine (1R), which share the same absolute configuration at the stereocenter but differ by inversion around the nitrogen atom. Table 1 summarizes the computed excitation energies together with the dipole and rotational strengths for the two analyzed diastereomers. Both the excitation energies and the relative intensities of the two singlet transitions are consistent with the experimental gas-phase spectra.^80^ For both transitions to *S*_1_ and *S*_2_, the rotational strength reverses sign upon inversion around the nitrogen atom. For the first transition, 1S exhibits a positive rotational strength, whereas 1R is negative. For the second transition, the same trend is observed. This behavior is consistent with the picture put forward by Rauk,^79^ and indicates that the handedness of the low-energy chiroptical response follows the nitrogen configuration, with the carbon stereocenter playing a comparatively spectator role. The systematic inversion of the rotational strength upon changing only the nitrogen stereochemistry supports the conclusion that the sign of the low-energy ECD transitions is primarily controlled by the nitrogen center, while the fixed carbon stereocenter modulates the magnitudes of the excitations without dictating their signs. However, the contribution of the stereocenter with respect to the nitrogen inversion in the second transition remains unclear. This motivated the TCD analysis of the *S*_0_ → *S*_2_ transition presented below, which aims to clarify where the chiroptical response is localized in real space.

**Table 1:** Excitation Energies (*E*, in eV, and λ, in nm), Dipole Strength (DS, 10^–36^ cgs), and Rotational Strength (RS, 10^–40^ cgs) of the First Two Singlet Excitations of the 1S and 1R Diastereomers of 2-Methylaziridine and of the First Excitation of the Corresponding Radicals

diastereomer	transition	*E*	λ	DS	RS
1S	*S*_0_ → *S*_1_	6.12	202.60	0.15	6.33
1R	*S*_0_ → *S*_1_	6.19	200.21	0.50	–9.39
1S	*S*_0_ → *S*_2_	6.87	180.59	0.65	–5.87
1R	*S*_0_ → *S*_2_	6.63	187.12	0.38	10.90
1S	*D*_0_ → *D*_1_	3.03	408.96	0.02	18.30
1R	*D*_0_ → *D*_1_	3.39	365.27	0.02	–11.16

As a first step, we computed the interconversion barrier and examined the evolution of the TCD maps for the *S*_0_ → *S*_2_ excitation in response to the NH large-amplitude motions. Figure 5 reports the energy profiles together with the TCD representation at five points along the displacement coordinate. From left to right, the 1S configuration converts into the 1R configuration. Several rearrangements in the electronic current can be observed. Most notably, the TCD field for 1R exhibits a well-structured circulation of charge, which progressively decreases in intensity as the system approaches the transition state (TS), and is almost absent in the (1*S*,2*S*) configuration. A more detailed analysis reveals that for 1S, the current pattern is comparatively fragmented, with sizable local circulations along the CH_2_ moiety and the methyl group, as well as in the nitrogen center. The current does not form a single dominant circulation about the N–H fragment, but instead appears as a more fragmented pattern with non-negligible components directed along the C–N bonds and into the surrounding space, such that the nitrogen-centered circulation is not predominant. At the same time, the contribution associated with the carbon stereocenter is comparatively weak, suggesting that it does not contribute appreciably to the *S*_0_ → *S*_2_ transition current.

**Figure 5:**
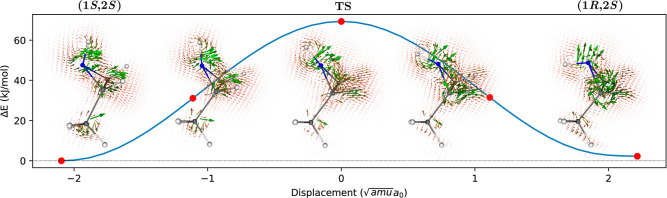
Interconversion path between (1*S*,2*S*) and (1*R*,2*S*)-2-methylaziridine. The insets show the TCD for the *S*_0_ → *S*_2_ excitation. All steps are shown with identical arrow scaling to allow direct comparison.

In contrast, for the 1R diastereomer, the current forms a well-defined, closed circulation around the N–H fragment, consistent with a dominant component localized at the heteroatom and its immediate bonding environment. The carbon-centered loop around the methyl fragment remains present, but it is less dominant relative to the nitrogen-centered circulation, and the overall current pattern appears more coherently organized over the C–C–N skeleton. The stereocenter manifests a more intense TCD pattern and contributes largely to the chiroptical response.

Overall, these TCD maps indicate that an inversion involving the nitrogen atom reorganizes the dominant circulation in its vicinity and modulates the local currents in the carbon framework. This real-space picture supports the conclusion that the low-energy chiroptical response is primarily governed by nitrogen inversion, while the fixed carbon stereocenter plays a secondary role by modulating the intensity of the ECD signal.

As a further application, we assessed whether inversion at the nitrogen atom also impacts the radical species. This point is particularly relevant because, in the closed-shell system, the HOMO corresponds to the nitrogen lone-pair orbital. Accordingly, the lowest electronic excitation, *D*_0_ → *D*_1_, was analyzed for the two radical diastereomers. In both radicals, the first transition involves the β-SOMO and the three nearest occupied β orbitals (SOMO-1_β_ to SOMO-3_β_). Table 1 reports the corresponding *D*_0_ → *D*_1_ excitation energy, as well as dipole and rotational strengths. Although the dipole strengths are comparable for the two radicals, the rotatory strength shows a sign inversion, which can be rationalized through the TCD maps. Figure 6 reports the TCD patterns for the lowest transition. Notably, the radicals exhibit a more delocalized current pattern over the ring, indicating a more diffuse response of the C–N–C ring in the open-shell excitation. For the 1S radical, the current is broadly distributed over the three-membered ring and displays a toroidal component emerging from the ring plane, primarily involving the C–N–C framework. An additional contribution arises from the methyl carbon. However, it remains spatially localized and does not perturb the ring-centered toroidal pattern. In the 1R radical, the direction of the toroidal current is reversed, whereas the localized methyl-carbon contribution remains unchanged. Again, the TCD analysis supports the conclusion that the sign inversion of the optical activity is governed by the nitrogen center.

**Figure 6:**
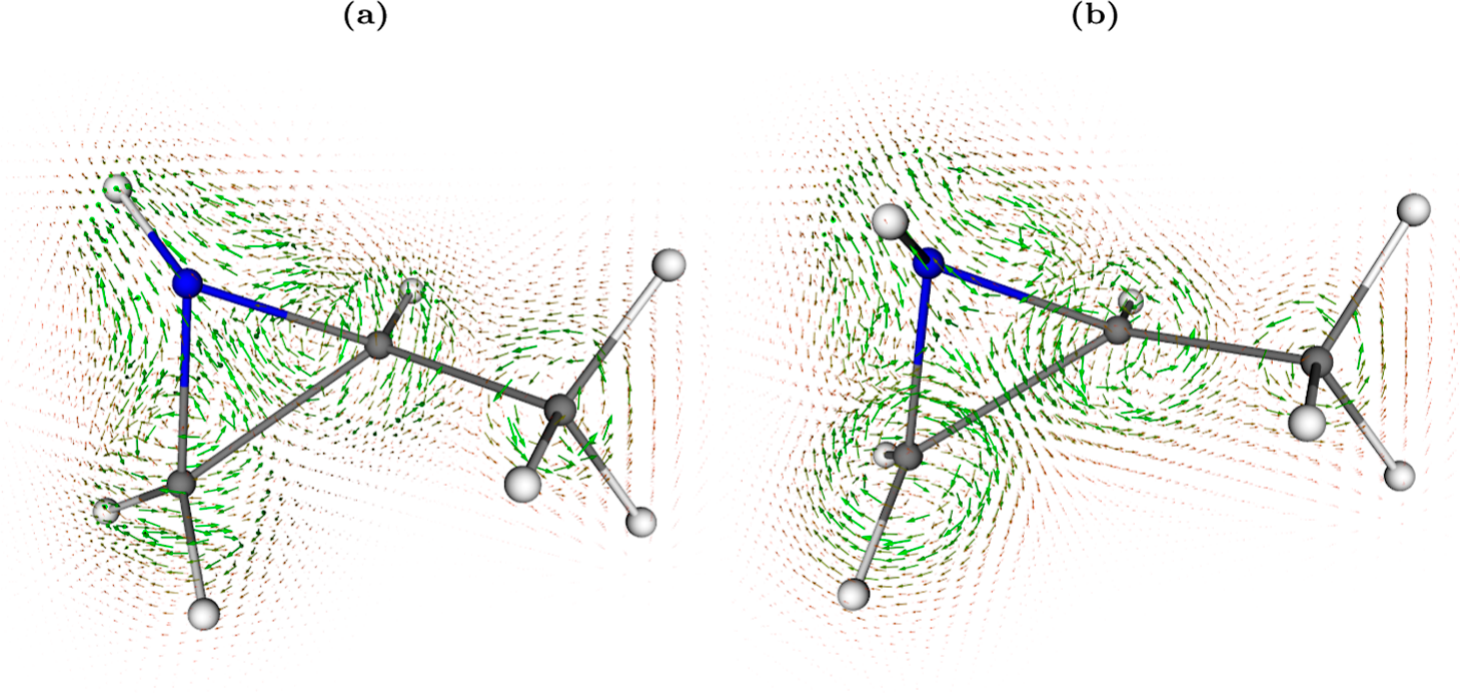
TCD maps for the *D*_0_ → *D*_1_ excitation of the (a) 1S and (b) 1R radicals. Both panels are shown with identical arrow scaling to allow direct comparison.

As a second example, we consider the alleno-acetylenic crown macrocycle (P,P,P,P)-(−)-1, whose chiroptical properties were studied in ref 69. In that study, the authors showed that the lowest–energy transition—associated with intense ECD signals—arises from electronic excitations characterized by a small EDTM and a large MDTM, resulting in an extraordinarily strong Cotton effect. Their interpretation was based on the analysis of the topology and symmetry of the molecular orbitals (MOs) involved in these transitions. Here, we revisit this system by computing and visualizing the TCDs associated with the two lowest ECD transitions in order to complement the MO-based picture with a real-space description of the electronic flow. This representation is expected to provide a more direct and intuitive insight into the origin of the chiroptical response.

As shown in Table 2, for the lowest–energy transition, the EDTM is extremely small and directed along the *z* axis, chosen here to coincide with the C_4_ axis of the molecule, which belongs to the D_4_ point group. The MDTM, instead, reaches a much larger value and is antiparallel to the EDTM, in full agreement with the strong negative Cotton effect reported in ref 69. In Figure 7 (left panel), the TCD reveals four current flows along the butadiyne segments of the macrocycle, arranged in a circular fashion around the crown. Their in-plane components cancel largely by symmetry, leaving only a small residual contribution along the *C*_4_ symmetry axis that accounts for the weak net EDTM. Conversely, strong current loops are observed around the four allene moieties. The 2D plot associated with **j**_01_^ang^(**r**) (Figure 7, right panel) reveals the origin of MDTM, the nearly continuous ring current along the macrocyclic ring that follows the four C≡C–C≡C segments that give rise to coherent contributions oriented along the *C*_4_ symmetry axis. The intense circulation of charge observed in the **j**_01_ plot, centered on the allenes, however, cancels out, giving no contribution to MDTM. This topology fully explains the large MDTM found along the *C*_4_ symmetry axis.

**Table 2:** Excitation Energies (E, in eV), EDTM and MDTM Components (in au) for the First Two Singlet Excited States of the Alleno–Acetylenic Crown Macrocycle (P,P,P,P)-(−)-1

transition	*E*	μ_0n,*x*_^el^	μ_0n,*y*_^el^	μ_0n,*z*_^el^	*m*_n0,*x*_^el^	*m*_n0,*y*_^el^	*m*_n0,*z*_^el^
*S*_0_ → *S*_1_	3.472	0.000	0.000	–0.208	0.000	0.000	2.145
*S*_0_ → *S*_2_	3.511	0.000	0.000	0.002	0.000	0.000	–0.009

**Figure 7:**
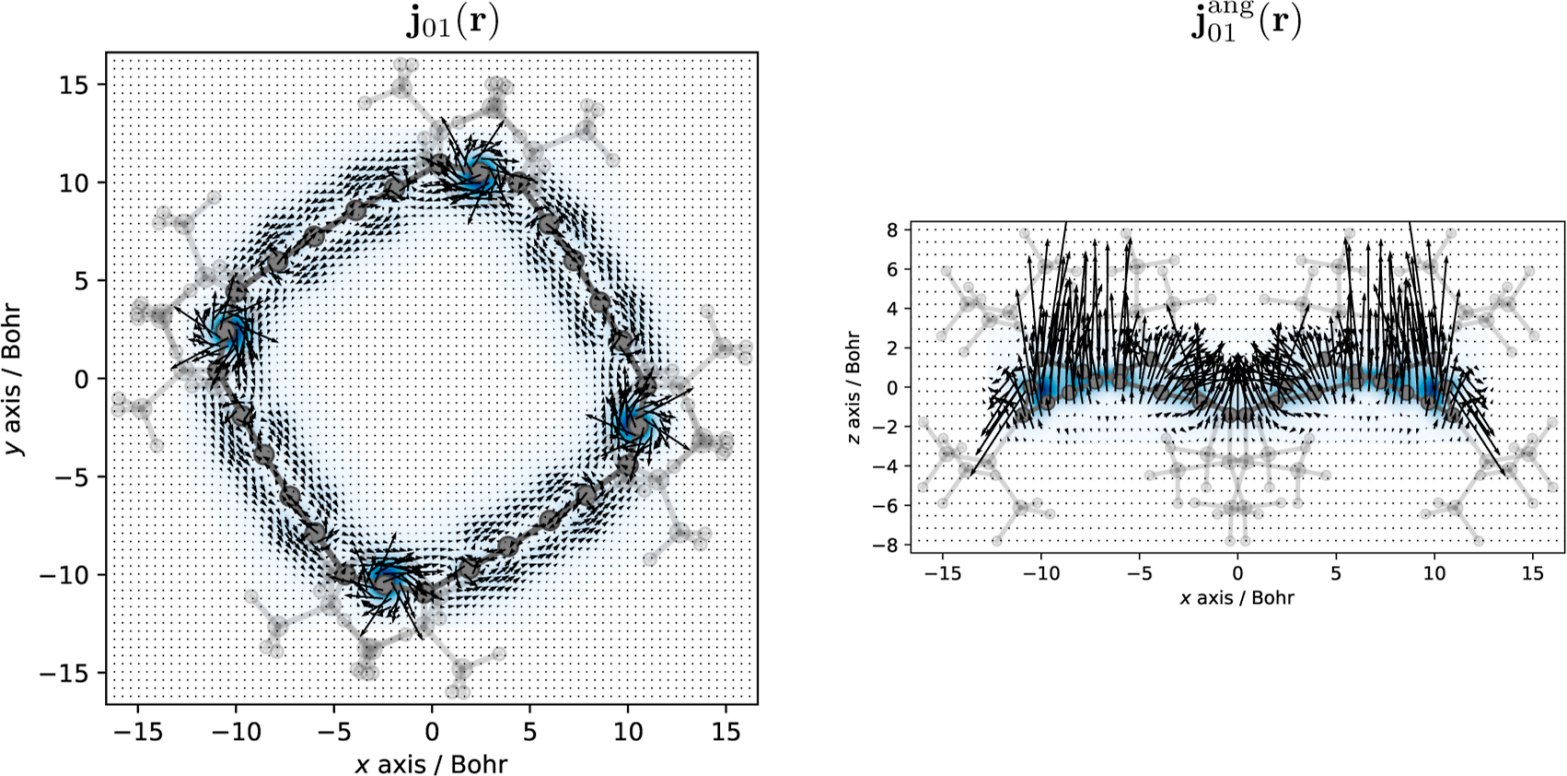
2D quiver plots of TCD and angular TCD for the first electronic transition. For clarity, vectors with magnitude greater than 0.5 au were truncated.

The transition to the second excited state shows a markedly different pattern. Both EDTM and MDTM are much smaller in magnitude than for the first transition (Table 2). The TCD associated with the EDTM (Figure 8, left panel) is now predominantly located on the butadiyne segments, with a weaker pattern on the adjacent allene units. The observed anisotropic polarization of the electronic flow is consistent with the MO analysis from ref 69, where the origin of this transition was attributed to excitations localized on specific pairs of butadiyne and allene fragments rather than delocalized uniformly around the ring. The TCD associated with the MDTM (eq 5), shown in Figure 8 (right panel), still displays several contributions distributed along the whole system but, without a common orientation, they cancel each other out by symmetry, in line with the smaller MDTM and the weaker contribution to the overall ECD spectrum.

**Figure 8:**
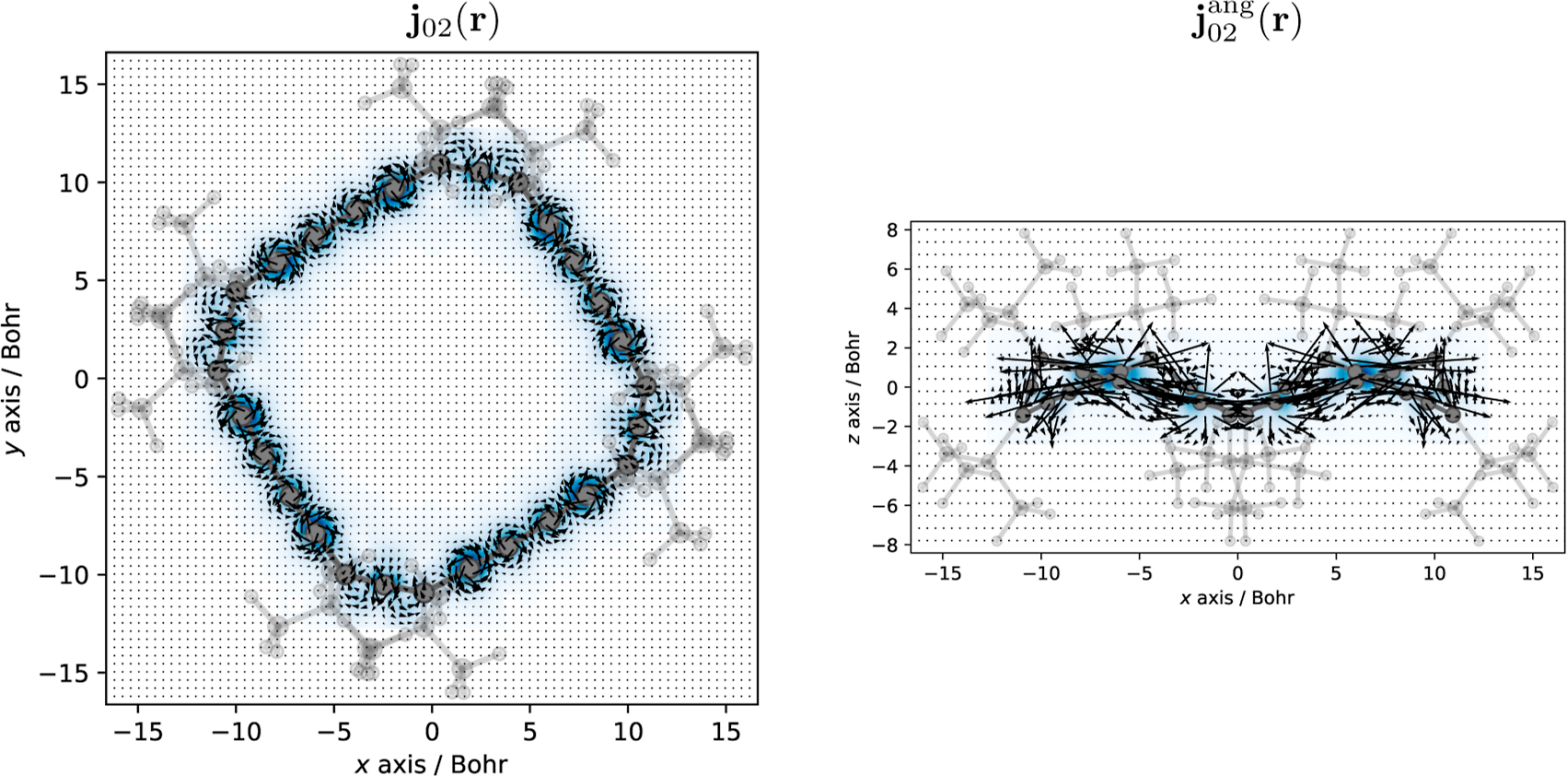
2D quiver plots of TCD and angular TCD for the second electronic transition. For clarity, vectors with magnitude greater than 0.5 au were truncated. **j**_02_^ang^(**r**) arrows were scaled by 0.5.

The crown macrocycle, with its different regular segments, represents an excellent test case of partitioning the current between molecular regions. Individual atomic contributions can be assessed by assigning volumetric space points to fragments, whose integrated current yields the contribution of the fragment to the EDTM and, in the case of the angular current, to the MDTM. For partitioning, we employ the AIM scheme.^81,82^ Figure 9a reports the partitioning for the *S*_0_ → *S*_1_ transition. The *tert*-butyl groups, which contribute only marginally to the transition, were excluded from the analysis. The AIM scheme identifies eight regions: four associated with the less extended butadiyne units and four associated with the allene units, the latter providing a markedly larger contribution.

**Figure 9:**
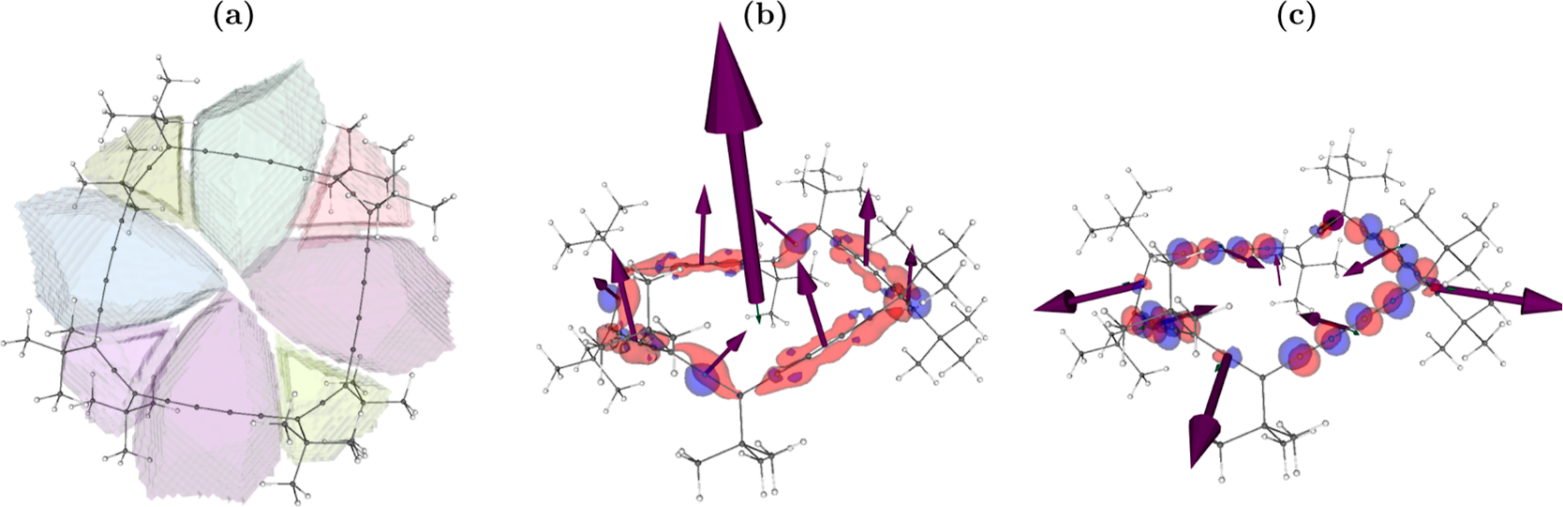
(a) Representation of the surfaces enclosing the partitions of the cube in the real space; the colors are chosen to differentiate better each fragment. In panels (b) and (c) the *R*_0*n*_^*m*^(**r**) isosurfaces for the first and second electronic states are shown: blue lobes represent positive contributions to ECD signal, and red the negative ones. The arrows represent the overall contributions to the EDTM (green) and MDTM (purple) of each fragment, centered at its center of mass.

In panels b and c of Figure 9, the contributions to the MDTM are depicted as purple vectors located at the center of each fragment, while the total transition dipoles are displayed at the center of mass of the system. Consistent with the TCD analysis, for the first transition, all portions of the ring contribute to the total transition dipole, with the largest contributions arising from the C≡C–C≡C segments. Diversely, the second transition shows also contributions in the allene regions. However, these contributions are antiparallel, producing a very small rotational strength.

For an easier view of the contributions from the different molecular segments, we also show in Figure 9b,c the scalar field *R*_0*n*_^*m*^(**r**) introduced in eq 17. Red isosurfaces represent negative contributions to the total rotational strength of the system, whereas blue isosurfaces represent positive contributions. Once again, the first transition produces coherent contributions along the entire macrocycle, whereas the second transition exhibits an alternating pattern within each molecular segment, resulting in an overall negligible rotational strength.

### 5.2 Transition Current Densities for Vibrational Circular Dichroism

As the first application to VCD, we examine α-phenylethylamine, studied in ref 71, where matrix-isolation infrared (IR) and VCD spectra in Ar and N_2_ at low temperature were supported by DFT calculations to analyze the packing effects of matrix environments on the molecular structure and resulting signal. Under matrix-deposition conditions, efficient conformational cooling yields IR spectra in both matrices consistent with a single conformer. However, the matrix VCD spectra deviated significantly from the gas-phase predictions for the main conformer, hinting at possible matrix-induced distortions within the same energy valley. A study of the conformational landscape, focused on the torsion of the phenyl group, was carried out, with the potential energy surface featuring several conformers separated by low barriers and, notably, a very shallow well around the global minimum (hereafter c1). By comparison of VCD spectra at distorted geometries with experimental data, the authors concluded that, in Ar, the matrix constraints led to a rotation of the phenyl ring with respect to the c1 minimum, whereas in N_2_, the distortion was characterized by a combination of phenyl rotation and torsion of the amino group. Although these structural changes are modest, they significantly affect the VCD pattern while leaving the IR spectrum largely unchanged. Intrigued by these conclusions, we have chosen to carry out a complementary theoretical analysis focused on phenyl rotation around the c1 minimum, considering the gas-phase c1 structure and two representative distorted structures, c1_*p*35_ and c1_*m*35_, obtained by rotating the phenyl ring by +35° and −35°, respectively, about the N–C*–C_1_–C_2_ dihedral, depicted in Figure 10. The configurations were chosen based on the predicted VCD spectra, selecting structures with notable differences in the band shape.

**Figure 10:**
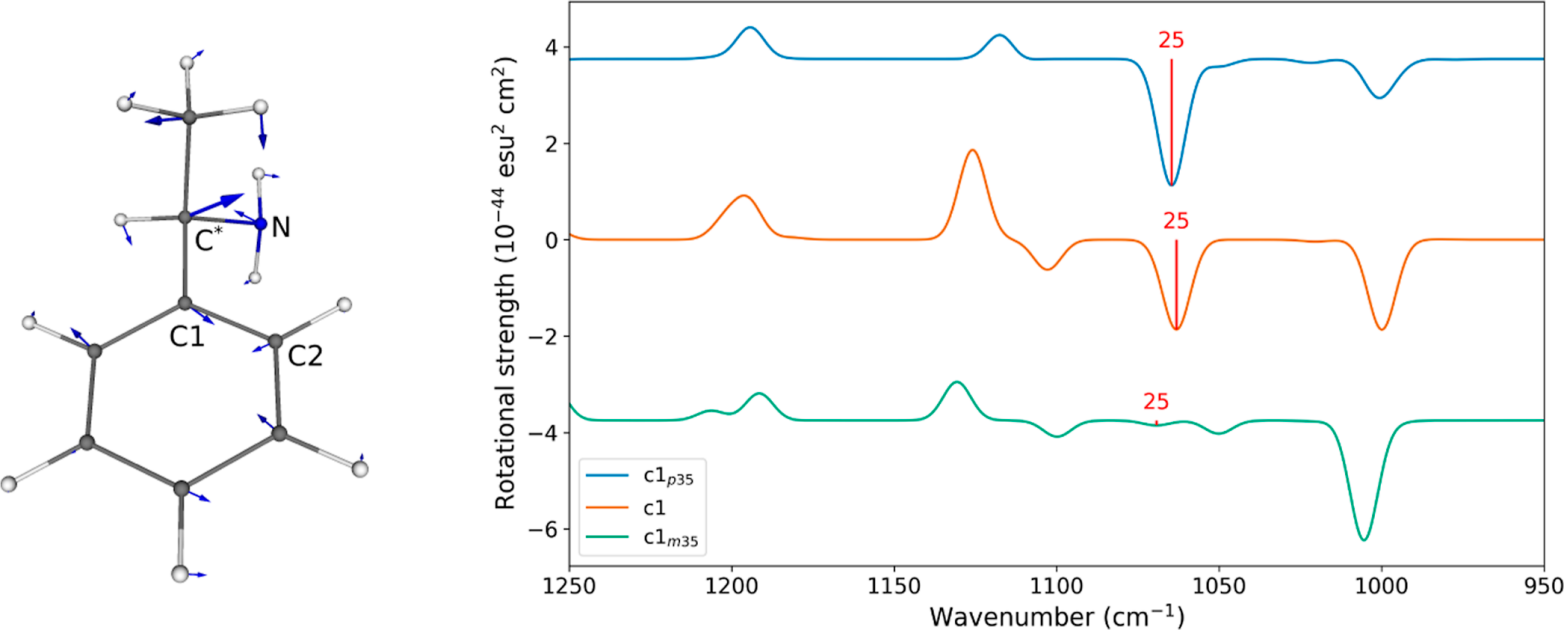
On the left, graphical representation of the dihedral angle considered in the VTCD analysis of the PEA c1 conformer and nuclear displacements for normal mode 25 of the PEA c1 conformer. On the right, VCD spectral region from 950 to 1250 cm^–1^ of the PEA c1_*p*35_, c1, and c1_*m*35_ molecules. The line corresponding to the fundamental transition of mode 25 is also displayed in red. The full width at half-maximum is set to 10 cm^–1^, using Lorentzian distribution functions for the empirical broadening.

To rationalize the changes in the different conformations, we examined the band related to the fundamental transition of mode 25 (Figure 10), which was found in ref 71 to be particularly sensitive to the phenyl rotation. Because a vibrational transition involves both nuclear and electronic contributions to the transition dipole moments, which often point in opposite directions and thus partially cancel one another, we first analyzed the normal-mode displacement pattern, as it provides an immediate qualitative indication of the nuclear component. The displacement vectors associated with normal mode 25 in the two distorted structures are shown in Figure S2 in the Supporting Information. In both c1_*p*35_ and c1_*m*35_ the motion is largely concentrated in the side chain, involving the stereogenic carbon, the C*–C_Ar_ bond and the amino group, with only smaller displacements on the phenyl ring. Thus, mode 25 can be qualitatively described as a mixed deformation of the chiral center and of the C*–C_Ar_ bond, weakly coupled to a collective distortion of the aromatic fragment. However, in c1_*p*35_ (on the right of Figure S2 in the Supporting Information), the displacements of the stereogenic carbon and its directly bonded phenyl carbon are almost in phase, so that the C*–C_Ar_ fragment moves as a nearly rigid unit and the chiral center position with respect to the aromatic ring is only weakly distorted. By contrast, in c1_*m*35_ (on the left of Figure S2 in the Supporting Information), the displacement vectors of these two atoms form a much larger relative angle, effectively introducing a stronger perturbation of the local chiral environment. The electronic contribution to the chiroptical response can be analyzed with the VTCD maps. However, since the VTCD expression in eq 15 is written as an infinite sum over electronic states, it must be truncated in practice. Thus, we first examined the behavior of the NACs as a function of the excitation energy for the c1 conformer. Figure 11 reports the excitation energies and NAC values for the lowest 300 singlet excited states. Excitation energies increase smoothly with the state index, whereas the NACs fluctuate around zero with several pronounced peaks distributed over the whole manifold of states. Thus, individual high-lying contributions cannot be strictly neglected. The energy denominators provide only a moderate attenuation, while occasional large NACs can display large individual terms.

**Figure 11:**
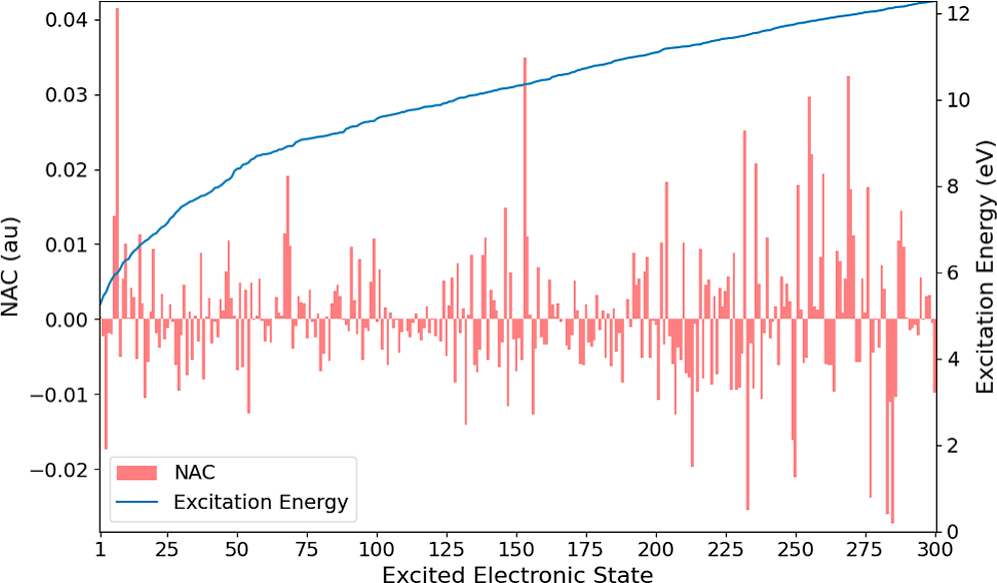
Excitation energies (blue line, right *y* axis) and corresponding NACs (red bars, left *y* axis) for the lowest 300 singlet excited states of the c1 conformer of PEA.

Since no clear term-based criterion is available to assess the convergence of the sum-over-states expression in eq 15, we examined how the observed TCD pattern changes upon inclusion of different numbers of excited states, as shown in Figure S3 of the Supporting Information. Such an approach is consistent with our aim of providing a qualitative description to aid the interpretation of the electronic contribution to the observed signals, rather than a quantitative evaluation of VCD intensities. A clear evolution of the current density is observed, with increasing contributions to **J**_0*k*_(**r**) on the asymmetric carbon and on the methyl group. The results obtained with 50 and 100 excited states are evidently incomplete, as some portions of the molecule involved in the vibration display current patterns that differ substantially from those obtained with 300 states. Between 150 and 200 states, all characteristic features begin to emerge, although some contributions still appear underestimated. These patterns remain largely stable upon increasing the number of states up to 300, where only an enhancement of the relative current intensity in specific regions of the molecule is observed. These observations suggest that, for similar systems, such as those involved in conformational analyses, a qualitative comparison of the current density **J**_0*k*_(**r**) computed by including in the summations the same number of excited states can already provide valuable structural insights, even in the absence of full convergence. Thus, since adding more states would substantially increase the computational cost without much improvement, we retain the lowest 300 singlet states as a practical compromise and adopt this truncation throughout, focusing primarily on the qualitative interpretation of the resulting VTCD maps.

The VTCD representations for both c1_*p*35_ and c1_*m*35_ are shown in Figure 12, respectively. For c1_*p*35_, the VTCD map reported in Figure 12a exhibits a clearly localized pattern. The most intense currents are distributed in the region of the chiral center and the amino group, while the phenyl ring is mainly surrounded by weaker and more diffuse contributions. This distribution gives rise to a coherent chiral flow primarily along the side chain with only minor compensation from the aromatic ring. By contrast, the VTCD for c1_*m*35_ in Figure 12b reveals a more balanced pattern, with currents of comparable magnitude distributed between the phenyl ring and the side chain. Domains are clearly visible around the aromatic moiety and in the region of the chiral center, so that positive and negative contributions to the rotational strength tend to cancel out upon spatial integration. Overall, for mode 25, these analyses show that rotating the phenyl ring switches the balance between constructive and destructive chiral current patterns in the two distorted molecules, thereby tuning the corresponding VCD response from strongly active to nearly silent. These results indicate that the phenyl system does not behave as a passive moiety but rather as an active modulator of the chiroptical response. In the c1_*p*35_ configuration, it cooperates with the stereogenic center and thus enhances the VCD signal, while in c1_*m*35_ it competes with it and effectively dampens the overall response. This confirms that the VCD intensity is extremely sensitive to the relative spatial arrangement between the chiral center and the aromatic ring.

**Figure 12:**
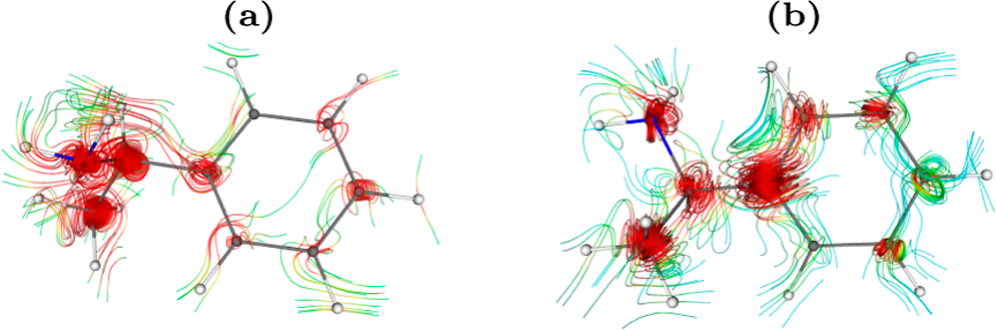
Computed VTCD associated with the fundamental of mode 25 of PEA for (a) c1_*p*35_ and (b) c1_*m*35_. Colors indicate the magnitude of the current density (blue: low; red: high).

In Table 3, the total, electronic, and nuclear EDTMs and MDTMs for the fundamental transition of mode 25 in the three PEA structures are reported. In all cases, the total EDTM is markedly smaller than the electronic and nuclear contributions, reflecting a cancellation due to the near-antiparallel alignment of the electronic and nuclear EDTMs. Moreover, the direction is governed by the nuclear EDTM because of its larger magnitude with respect to the electronic counterpart. In contrast to the EDTM, the MDTM displays a pronounced conformational dependence in its orientation. While the total magnitudes are of comparable value across the distorted structures, the direction of the total MDTM changes substantially. For c1_*p*35_, its main contributions are along *y*- and *z*-axis. The decomposition further reveals that the *z* component is essentially electronic, whereas the *y* component is almost entirely nuclear. For conformer c1, the total MDTM has both electronic and nuclear terms that contribute largely and with the same sign in the *z* component. Finally, for c1_*m*35_, the MDTM remains dominated by the *z* direction but with an opposite sign compared to c1. In the context of the matrix-isolation study carried out in ref 71, these results imply that even modest matrix-induced distortions can dramatically alter the VCD intensity of a given band while leaving the IR spectrum almost unchanged, confirming the observations made by the authors.

**Table 3:** Total, Electronic, and Nuclear EDTM and MDTM (in au) Corresponding to the Fundamental Transition of Mode 25 for the Three PEA Structures Examined

	μ_*x*_	μ_*y*_	μ_*z*_	μ	*m*_*x*_	*m*_*y*_	*m*_*z*_	*m*
**c1**_*p* **35**_								
tot	–0.019	0.102	0.035	0.110	0.044	–0.139	0.122	0.190
ele	0.105	–0.208	–0.237	0.332	–0.020	–0.000	0.121	0.123
nuc	–0.124	0.310	0.272	0.431	0.064	–0.139	0.001	0.153
**c1**								
tot	–0.020	–0.101	–0.024	0.106	0.068	0.014	0.203	0.214
ele	0.126	0.342	–0.066	0.371	–0.019	–0.034	0.098	0.106
nuc	–0.147	–0.443	0.042	0.469	0.087	0.048	0.104	0.144
**c1**_*m* **35**_								
tot	–0.003	0.092	0.045	0.103	–0.069	0.085	–0.186	0.216
ele	–0.169	–0.325	–0.045	0.369	0.027	0.005	–0.079	0.084
nuc	0.166	0.417	0.091	0.458	–0.096	0.080	–0.107	0.165

As a second example, we consider the Ru­(II) complex previously investigated in ref 72, a challenging case with a dense manifold of ligand-based vibrations and a well-characterized chiral response.^83^ The VCD spectrum of the metal complex, characterized by an intense and isolated CO stretching vibration, displays several intense bands in the fingerprint region, along with weaker features at lower frequencies dominated by collective metal–ligand vibrations (see Figure 13). For each normal mode considered in ref 72, and whose fundamental transition is marked in Figure 13, namely modes 22, 48, 61, 66, the corresponding VTCD was computed and rendered (the displacement vectors reported in Figure S4 in the Supporting Information).

**Figure 13:**
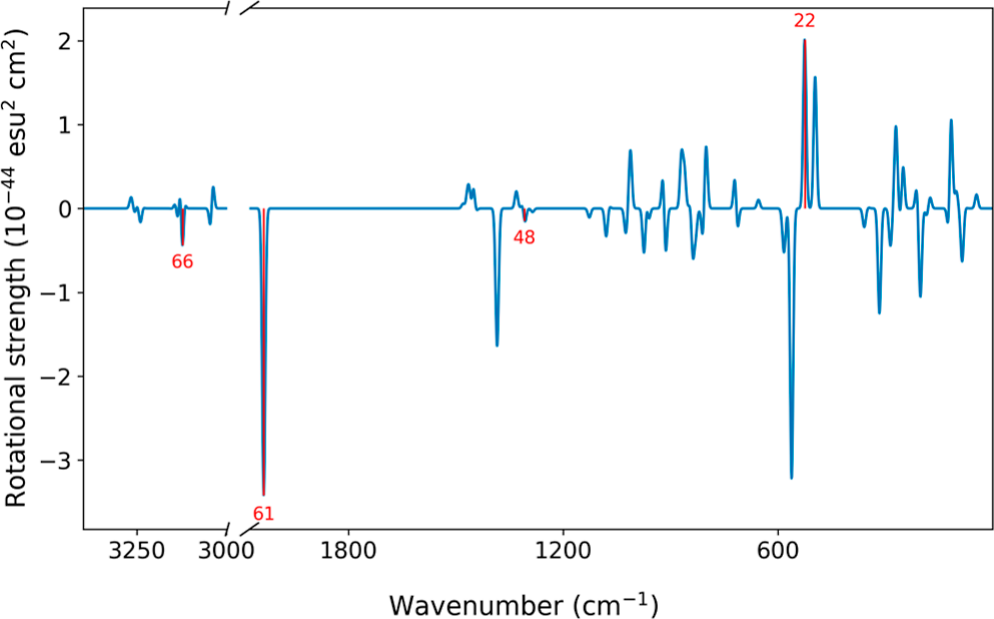
VCD spectrum of the Ru­(II) complex. The fundamental transitions of modes 22, 48, 61, and 66 are displayed in red. The full width at half-maximum is set to 10 cm^–1^.

We first analyzed the behavior of NACs along the excited state manifold to obtain a qualitatively reliable description of VTCD. Figure 14 shows the excitation energies and NACs as functions of the excited electronic states for the CO stretching mode.

**Figure 14:**
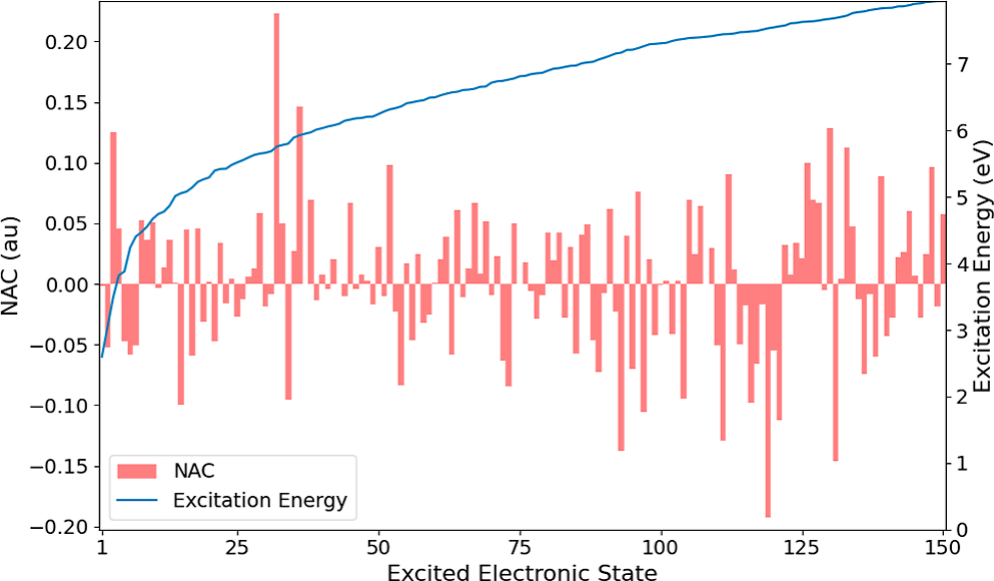
Excitation energies (blue line, right *y* axis) and nonadiabatic couplings, NAC (red bars, left *y* axis), as a function of the excited states for the CO stretching.

After the first 20 excited states, the excitation energies increase nearly monotonically across the manifold, and thus moderately suppress high-energy contributions. By contrast, the NACs oscillate around zero with sizable positive and negative peaks distributed over the full range of excited states, rather than being confined to the lowest ones. Similar trends for normal modes 22, 48, and 66 are reported in Figure S5 in the Supporting Information. Notably, modes 48 and 66 show more appreciable NAC contributions at higher states.

Thus, as noted in the previous case, not only low-energy states can contribute significantly to the VTCD, so a simple truncation criterion based purely on the energy would likely fail in most practical cases.

The evolution of the current density upon increasing the number of states in the sum-over-states is reported for the four analyzed normal modes in Figure S6 in the Supporting Information. Analysis of these representations reveals two distinct classes of normal modes: those involving predominantly light atoms, whose molecular orbitals associated with the relevant bonds are expected to be more energetically stabilized, and those involving vibrations coupled to the CO group. The latter category displays a faster apparent convergence, with characteristic patterns already evident when only 50 excited states are included in the summation. By contrast, and consistently with what was already discussed for the PEA case, modes involving light atoms begin to show localized contributions to **J**_0*n*_(**r**) on the atoms participating in the vibration only when 100 states are incorporated, with further smaller changes observed upon extending the summation to 150 states. Although full convergence is likely still distant, these trends indicate that meaningful qualitative insights can nonetheless be extracted from the analysis. For these reasons, and considering the fact that including additional states would increase significantly the computational cost, we use 150 states as a practical compromise for generating the VTCD maps, which in this work are interpreted primarily at a qualitative level.

Figure 15a shows the VTCD associated with the fundamental of mode 22. This low-frequency mode corresponds to a collective deformation of the inner coordination sphere, in which the chelating ligands rock as a unit around the metal center. The VTCD pattern is dominated by an intense current that flows along the Ru–ligand bonds. The strongest currents are confined to the Ru atom, while only weaker, more diffuse currents reach the ligands. The currents exhibit a coherent helical circulation around the Ru atom rather than being confined to a single bond, indicating that mode 22 probes the chiral environment of the entire coordination sphere. By contrast, the outer parts of the ligands contribute marginally to the current, consistent with their more spectator-like role in the VCD response of this mode. Table 4 reports the total, electronic, and nuclear EDTMs and MDTMs. For mode 22, a strong electronic-nuclear cancellation arising from the near-antiparallel alignment of the electronic and nuclear EDTM is observed, with the net EDTM direction being primarily set by the nuclear component. Conversely, the contributions of the MDTM components are mostly constructive, yielding a total magnetic transition dipole moment that is prevalently dominated by the *z* component.

**Figure 15:**
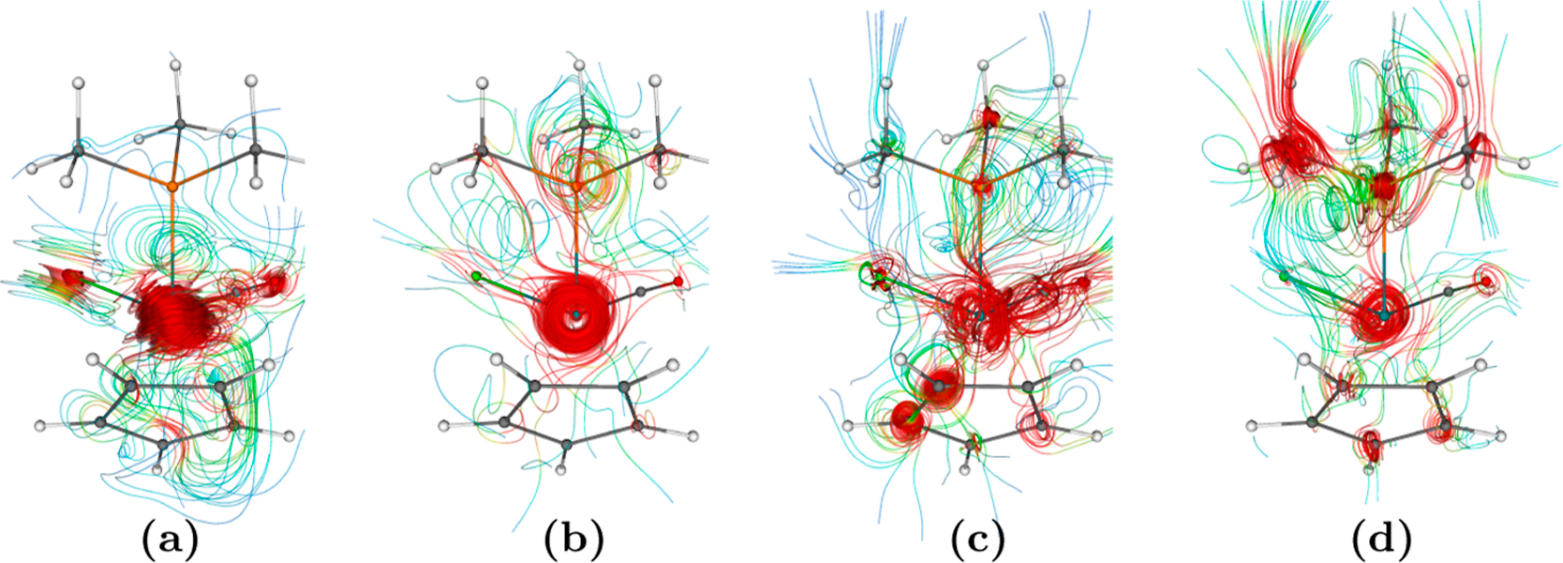
VTCD streamlines for the fundamental transitions of (a) mode 22, (b) mode 48, (c) mode 61, (d) mode 66 of the Ru­(II) complex. Colors encode the local magnitude of the current density (blue: low; red: high).

**Table 4:** Total EDTMs and MDTMs (in au), as Well as Their Electronic and Nuclear Contributions, for the Fundamental Transitions of the Selected Modes from the Ru­(II) Complex

	μ_*x*_	μ_*y*_	μ_*z*_	μ	*m*_*x*_	*m*_*y*_	*m*_*z*_	*m*
**Mode 22**								
tot	0.131	0.037	0.008	0.136	0.041	0.050	0.120	0.137
ele	–0.155	–0.047	0.070	0.176	0.036	0.051	0.050	0.080
nuc	0.285	0.084	–0.062	0.304	0.006	–0.001	0.070	0.070
**Mode 48**								
tot	0.101	0.005	–0.059	0.117	0.026	–0.036	–0.031	0.054
ele	–0.636	–0.068	0.248	0.686	0.118	–0.161	0.298	0.359
nuc	0.737	0.073	–0.306	0.802	–0.144	0.125	–0.329	0.380
**Mode 61**								
tot	–0.638	–0.078	0.668	0.927	–0.215	–0.014	–0.228	0.313
ele	–0.633	–0.079	0.659	0.917	–0.215	–0.013	–0.223	0.310
nuc	–0.006	0.001	0.009	0.011	–0.000	–0.001	–0.005	0.005
**Mode 66**								
tot	0.002	–0.111	0.020	0.112	0.245	0.070	0.096	0.272
ele	–0.066	–0.677	0.203	0.710	0.601	0.118	0.251	0.662
nuc	0.068	0.566	–0.183	0.599	–0.356	–0.048	–0.155	0.392

Figure 15b shows the VTCD associated with the fundamental of mode 48. The VTCD of this mode is markedly anisotropic and involves a subset of ligands. A high-intensity current is still found around the Ru center, but the strongest currents are now distributed along the metal–ligand axis associated with the P­(CH_3_)_3_ ligand. The streamlines reveal a well-defined loop that connects these ligands through the Ru atom, while only weaker, more diffuse currents are present on the remaining parts of the coordination sphere. This pattern indicates that mode 48 couples the environment of the Ru atom mainly through an asymmetric deformation of the P­(CH_3_)_3_ ligand, rather than through a fully collective rocking of the entire coordination sphere as in mode 22. The resulting VTCD thus reflects a more directional, ligand-focused contribution to the VCD response of the complex. For modes like 48, the electronic and nuclear contributions to both the EDTM and MDTM are large but nearly antiparallel, yielding a much smaller net total EDTM and MDTM (Table 4).

Figure 15c shows the VTCD associated with the fundamental of mode 61. This mode lies in the higher-frequency carbonyl stretching region and is dominated by the motion of the terminal CO ligand. Consequently, the VTCD pattern is strongly focused along the Ru–CO bond. These observations are also in line with the relatively isolated nature of CO vibrations observed at the anharmonic level as well.^72^ High-intensity currents emerge from the Ru atom and propagate along the Ru–CO bond, with only weaker currents in the remaining parts of the coordination sphere. Compared with modes 22 and 48, the current is clearly oriented through a single ligand, indicating that the chiral response of mode 61 is governed by the coupling between a largely localized CO stretch and the Ru coordination environment. The other ligands are less involved in the dominant CO-centered current loop; nonetheless, non-negligible currents are observed on the cyclopentadienyl ring. For mode 61, both the EDTM and the MDTM (Table 4) are largely defined by electronic rearrangements. The nuclear contributions are 2 orders of magnitude smaller than the corresponding electronic terms. As a result, the total EDTM and MDTM are essentially identical in both magnitude and direction to the electronic contributions. These characteristics make this vibration an ideal candidate for VTCD analysis. Indeed, the EDTM follows the linear charge flux oriented along the CO bonds, whereas the MDTM derives its intensity from the circulation of charge around the Ru center, thereby carrying information about its chiral environment.

Finally, Figure 15d displays the VTCD associated with the fundamental transition of mode 66. This mode lies in the high-frequency region dominated by C–H stretching motions. As expected, the VTCD pattern shows a more distributed current involving several ligand fragments. A high-intensity current is still present around the Ru atom, and yet strong current loops associated with the C–H stretches are now clearly visible on the methyl groups. The VTCD reveals multiple local vortices on these ligands that are connected through the metal, yielding a delocalized current pattern. Therefore, mode 66 couples a set of C–H-stretching motions distributed over the P­(CH_3_)_3_ ligand to the environment of the Ru atom, leading to more delocalized contributions to the VCD response. From a numerical perspective (Table 4), the EDTM exhibits pronounced electronic-nuclear cancellation, as they carry opposite signs across all the components, with the total EDTM being governed by its electronic component. The MDTM shows a similar compensation effect, with nuclear and electronic contributions opposing each other, leaving a reduced total MDTM that remains oriented toward the electronic MDTM. Nonetheless, this region should be interpreted with caution, as anharmonicity, both in its mechanical and electrical contributions, is known to play a crucial role.^72^

## 6 Conclusions

We have presented two new complementary tools, ELEMENTS and TCDLIBX, for the computation, rendering, and analysis of TCD and VTCD associated with ECD and VCD spectra. From standard quantum-chemical calculations, ELEMENTS can routinely generate and analyze three-dimensional current-density fields of any type of molecular systems, and provide a deeper understanding of ECD and VCD spectra beyond the simple prediction of the observables. When combined with the TCDLIBX visualization library, it is possible to directly process the generated maps and choose interactively the most suitable representation to further facilitate TCD and VTCD analyses.

The TCD implementation supports both closed- and open-shell systems and offers flexible strategies for grid generation and data reuse. The analysis presented here demonstrates that TCD and VTCD maps reveal qualitative differences in electronic structure and chiral response of the molecular systems that are not accessible from scalar observables alone. Beyond the examples discussed here, ELEMENTS is designed as a modular platform that can be easily extended to support additional electronic-structure methods and computational programs or to provide alternative response formalisms and more advanced analysis tools.

In the case of VTCD, the present implementation of ELEMENTS follows the VCT formalism and requires TD-DFT transition density matrices combined with NACs. This methodology presents practical limitations due to the reliance on the sum over all electronic excited states and the difficulty, illustrated in this work, to satisfactorily truncate it to reasonable levels. A natural next step is the implementation of a response theory formulation avoiding the sum-over-states approach, such as the magnetic-field perturbation theory (MFPT).^57^ Such an extension would enable the direct computation of current-density fields associated with response properties without explicitly constructing excited states, offering a more general and potentially more efficient route to VTCD, especially for large systems and dense manifolds of states.

More generally, the same framework can be extended to a broader class of molecular properties relevant for other spectroscopies. Examples include current densities associated with magnetic circular dichroism, as well as Raman and Raman optical activity signals. Future versions of ELEMENTS would thus explore the spatial origin of a wide range of spectroscopic signatures beyond ECD and VCD.

## Supplementary Material



## Data Availability

All 3D molecular representations present in this work were generated with the TCDLIBX library, available at https://github.com/mast-theolab/tcdlibx.git. The ELEMENTS code is available publicly, through its public GitHub repository at https://github.com/mast-theolab/elements.git and on Zenodo.^84^ All data used for the generation of the pictures related to the discussions and analyses can be found in a dedicated Zenodo repository at 10.5281/zenodo.20185671.

## Notes

Graphical comparison of two seed-placement strategies as a function of the number of seeds; visualization of the normal modes for PEA conformers and the ruthenium complex; evolution of TCD patterns with increasing number of states included in the sum-over-states for both PEA and the ruthenium complex; excitation energies and nonadiabatic coupling magnitudes as functions of the excited electronic state index for normal modes 22, 48, and 66 of the ruthenium complex (PDF)
