# Supplementary material for: Exploring the Origin of Molecular Chirality: A Standalone Suite of Tools to Visualize and Analyze Transition Current Density
Source: J Chem Theory Comput. 2026 Jun 24;22(13):6804–19. doi: 10.1021/acs.jctc.6c00980 (PMC13374041; doi:10.1021/acs.jctc.6c00980)
Supplement: Supplementary file 1 [file ct6c00980_si_001.pdf]

Supporting Information: Exploring the Origin of Molecular Chirality: a Standalone Suite of Tools to Visualize and Analyze Transition Current Density

Alberto Barlini,[*,†] Marco Fusè,[*,‡] and Julien Bloino[*,†]

†*Scuola Normale Superiore, Piazza dei Cavalieri 7, 56126 Pisa, Italy*

‡*Università degli Studi di Brescia, Dipartimento di Medicina Molecolare e Traslazionale, Viale Europa 11, 25123, Brescia, Italy*

E-mail: alberto.barlini@sns.it; marco.fuse@unibs.it; julien.bloino@sns.it

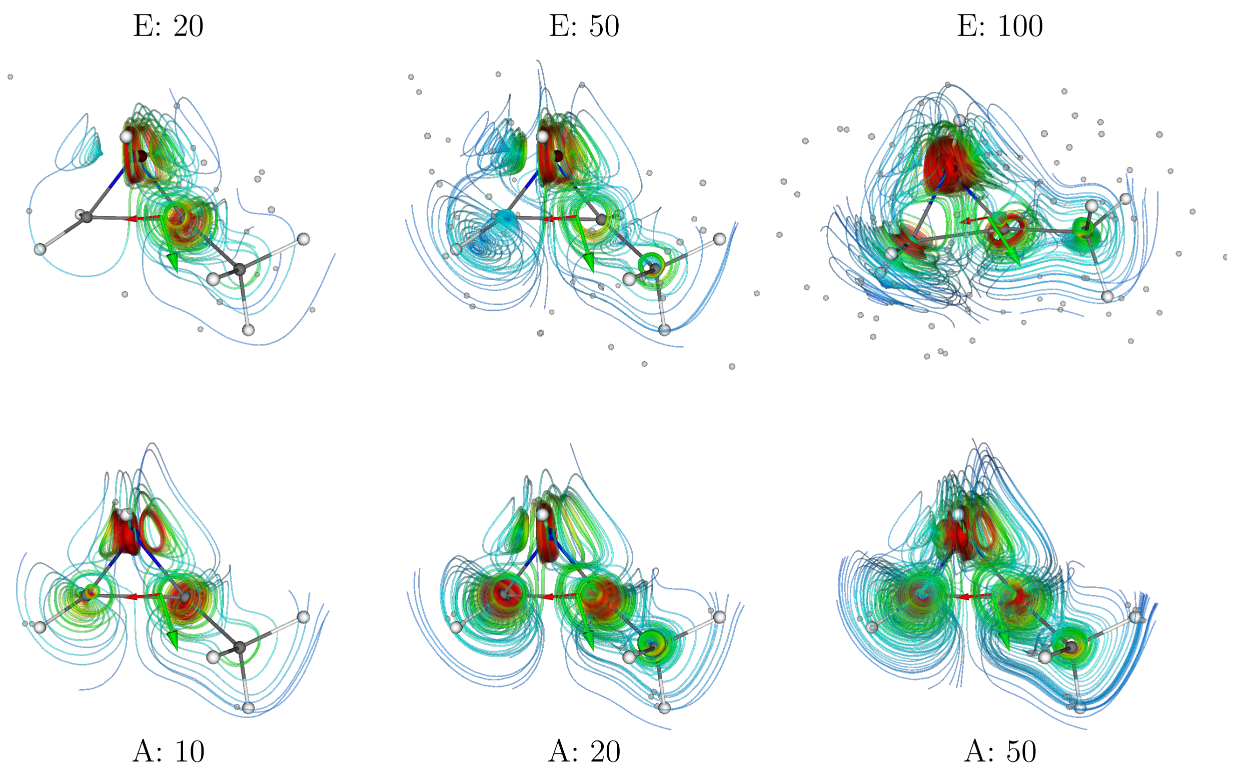

Figure S1: Comparison of streamlines representations of $\mathbf{j}(\mathbf{r})$ associated with the $S_0 \rightarrow S_1$ transition for the 2S-2-methylaziridine radical, obtained by varying both the number of seeds and the seeding strategy. In the figure, "E" and "A" denote seeds distributed within an ellipsoid or around the nuclei, respectively. All other representation parameters were kept fixed across the different visualizations.

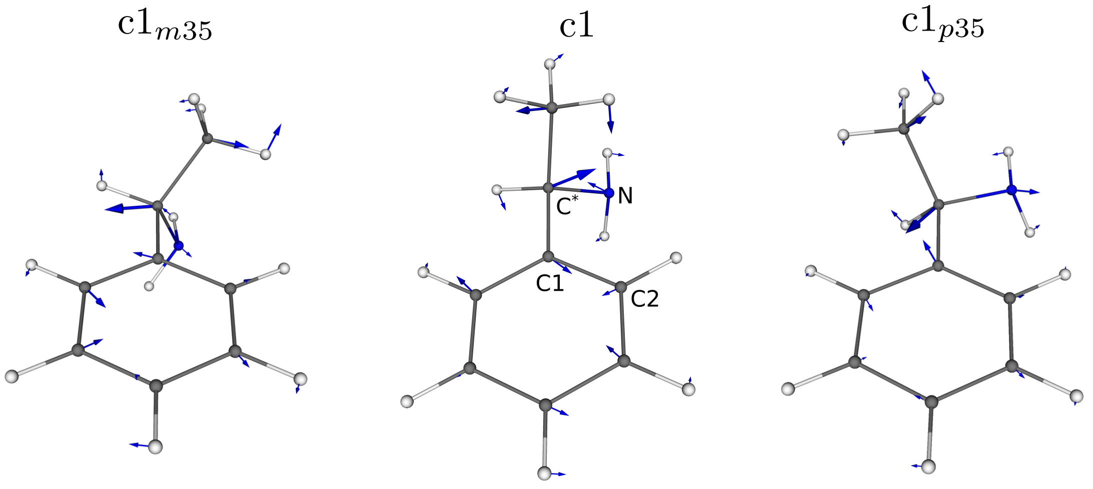

Figure S2: Nuclear displacements for normal mode 25 of PEA in c1$_{m35}$, c1 and c1$_{p35}$.

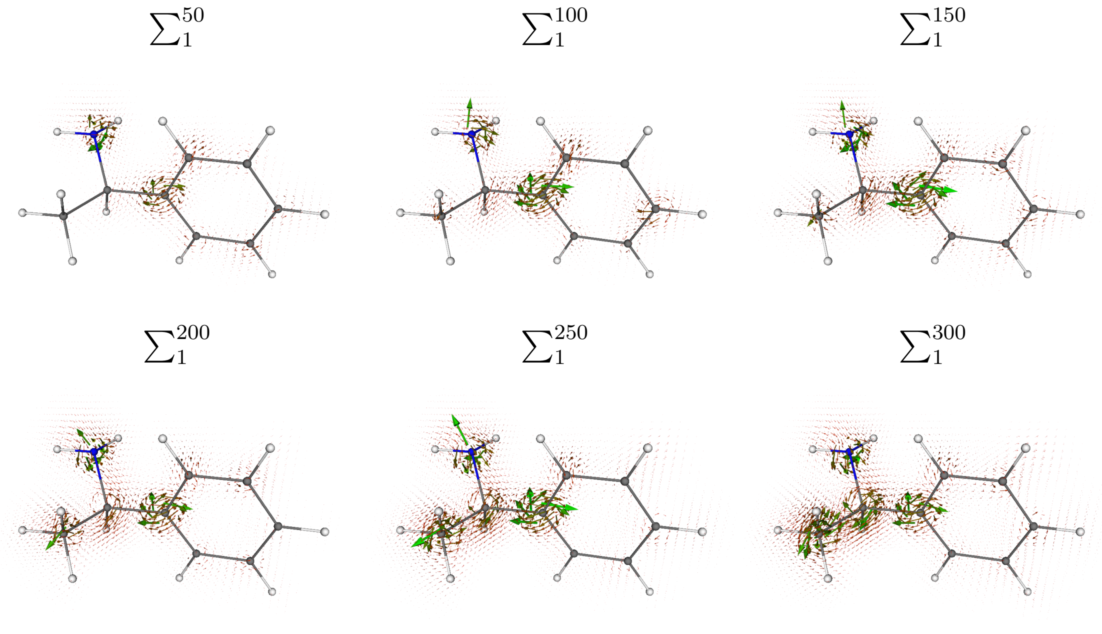

Figure S3: Computed $\mathbf{J}_{0n}(\mathbf{r})$ cubes with 50, 100, 150, 200, 250, and 300 electronic excited states relative to normal mode 25 of the PEA c1 conformer.

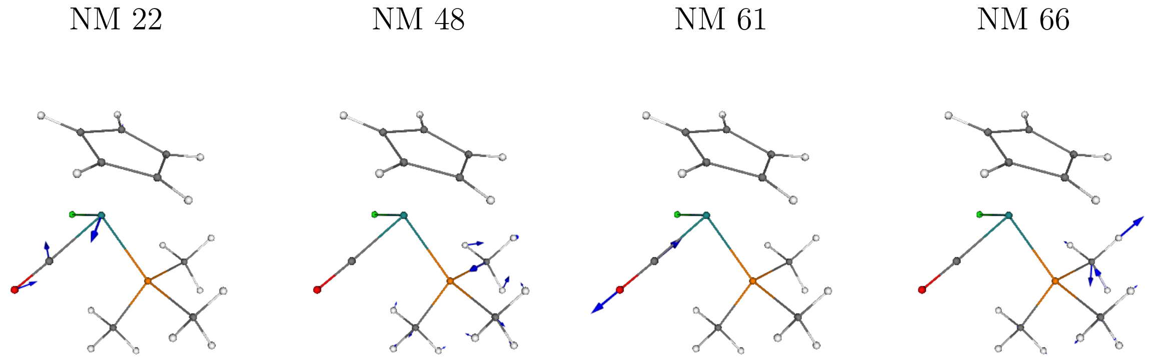

Figure S4: Nuclear displacements for normal modes 22, 48, 61, and 66 of the Ru(II) complex.

NM 66

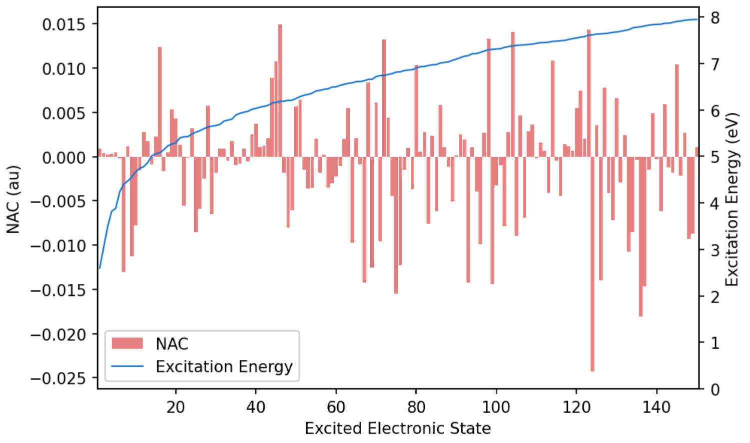

NM 48

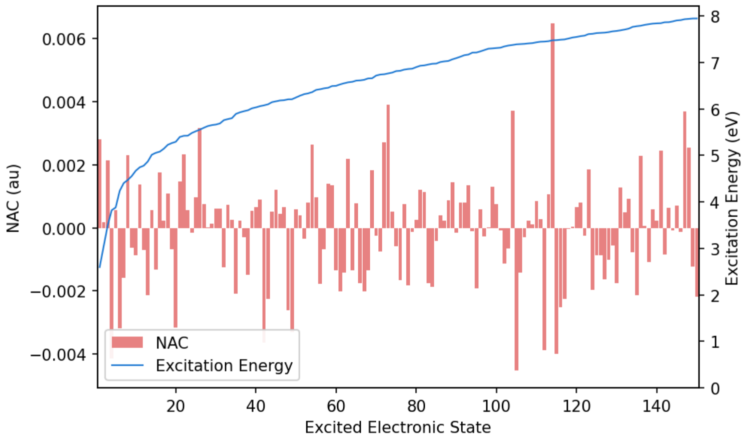

NM 22

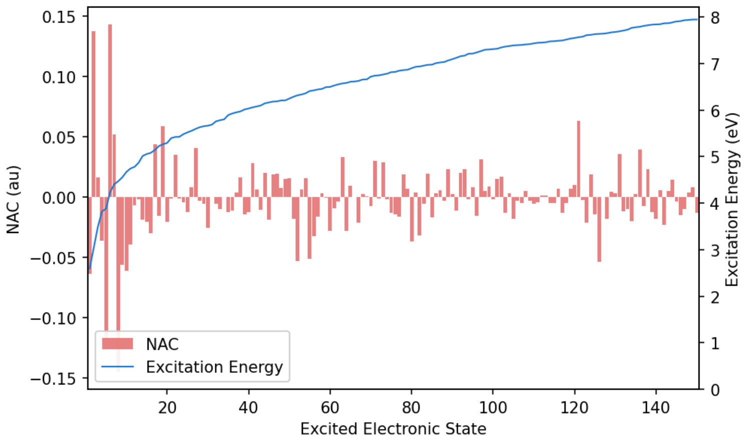

Figure S5: Excitation energies (blue line, right y axis) and nonadiabatic couplings, NAC (red bars, left y axis), as a function of the excited states for the NMs 22, 48 and 66.

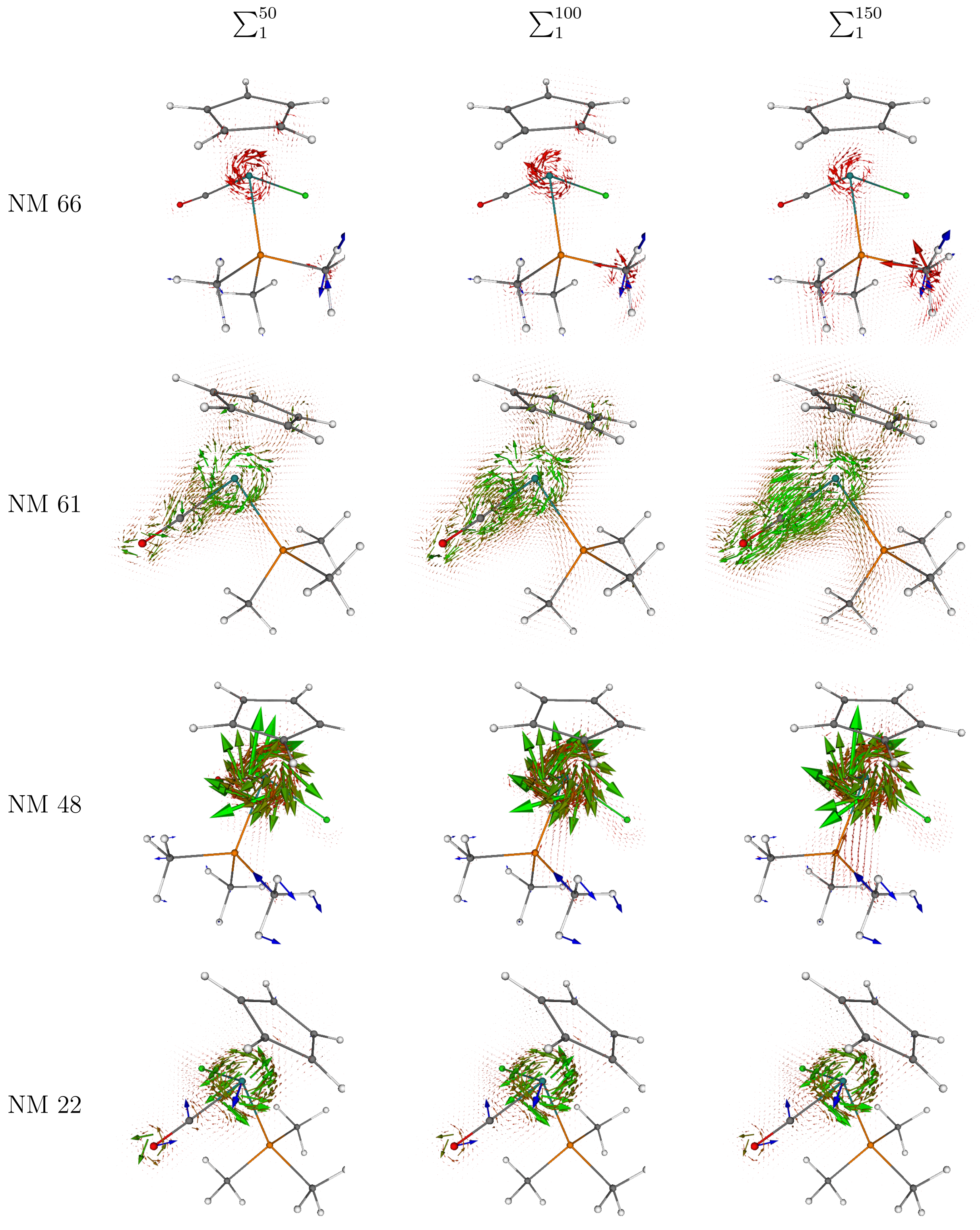

Figure S6: Computed $\mathbf{J}_{0n}(\mathbf{r})$ cubes with 50, 100, and 150 electronic excited states for the normal mode 22, 48, 61, and 66 of the Ru(II) complex. Volumetric datasets were sub-sampled displaying one element every five, and visualization threshold were tailed for each NM and kept constant across the different up points.
